# Nanotherapies Based on ROS Regulation in Oral Diseases

**DOI:** 10.1002/advs.202409087

**Published:** 2025-01-30

**Authors:** Xin Luo, Yanli Zhang, Yuting Zeng, Dehong Yang, Zhiyan Zhou, Ziting Zheng, Ping Xiao, Xian Ding, Qianlin Li, Jiaping Chen, Qianwen Deng, Xincen Zhong, Sijie Qiu, Wenjuan Yan

**Affiliations:** ^1^ Department of Stomatology Nanfang Hospital Southern Medical University Guangzhou 510515 China; ^2^ Stomatological Hospital School of Stomatology Southern Medical University Guangzhou 510280 China; ^3^ Department of Orthopedics Spinal Surgery Nanfang Hospital Southern Medical University Guangzhou 510515 China

**Keywords:** endogenous and exogenous stimuli‐responsive, nanorobot, nanotherapies, nanozymes, oral disease, reactive oxygen species (ROS)

## Abstract

Oral diseases rank among the most prevalent clinical conditions globally, typically involving detrimental factors such as infection, inflammation, and injury in their occurrence, development, and outcomes. The concentration of reactive oxygen species (ROS) within cells has been demonstrated as a pivotal player in modulating these intricate pathological processes, exerting significant roles in restoring oral functionality and maintaining tissue structural integrity. Due to their enzyme‐like catalytic properties, unique composition, and intelligent design, ROS‐based nanomaterials have garnered considerable attention in oral nanomedicine. Such nanomaterials have the capacity to influence the spatiotemporal dynamics of ROS within biological systems, guiding the evolution of intra‐ROS to facilitate therapeutic interventions. This paper reviews the latest advancements in the design, functional customization, and oral medical applications of ROS‐based nanomaterials. Through the analysis of the components and designs of various novel nanozymes and ROS‐based nanoplatforms responsive to different stimuli dimensions, it elaborates on their impacts on the dynamic behavior of intra‐ROS and their potential regulatory mechanisms within the body. Furthermore, it discusses the prospects and strategies of nanotherapies based on ROS scavenging and generation in oral diseases, offering alternative insights for the design and development of nanomaterials for treating ROS‐related conditions.

## Graphical abstract

This review examines the role of reactive oxygen species (ROS) in oral disease pathology and the therapeutic potential of ROS‐based nanomaterials. It discusses their enzyme‐like properties, unique compositions, and how they modulate ROS dynamics for oral health. The paper also explores recent advancements in nanomaterial design, their customization for oral medicine, and the implications for ROS‐related condition treatments.

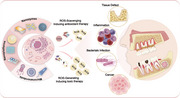

## 1 Introduction

Currently, the targeted regulation of intracellular Reactive Oxygen Species (ROS) levels is a hot topic in the treatment of oral diseases. The dynamic changes in ROS concentrations are critical factors in regulating the cellular redox homeostasis, playing a pivotal role in guiding various cellular activities such as proliferation, differentiation, migration, apoptosis, as well as for preserving tissue integrity and facilitating organismal repair.^[^ ^1^, ^2^ ^]^ Growing evidence indicates a close correlation between alterations in cellular redox homeostasis and the onset and severity of oral diseases.^[^ ^3^, ^4^, ^5^ ^]^ For instance, during periodontitis, the overproduction of ROS by cells disrupts redox homeostasis, exacerbating tissue damage and disease progression.^[^ ^6^ ^]^ Moreover, the oral cavity, being one of the most crucial contact environments in the human body, is frequently subjected to external influences such as bacterial infections, food debris, and chemical substances. Sustained exposure to these factors can modify the sensitivity and responsiveness of oral tissues (teeth, gums, mucosa, etc.) to ROS, adversely affecting oral health.^[^ ^7^, ^8^, ^9^ ^]^ Consequently, targeting the regulation of ROS levels may serve as a feasible strategy to ensure normal physiological functions in the oral cavity and reduce disease incidence. Currently, numerous approaches targeting ROS levels regulation, including metal ions, signaling molecules, and drugs, have been investigated.^[^ ^10^, ^11^ ^]^ However, these conventional drugs may encounter limitations in clinical applications, such as short circulation times, lack of controlled release characteristics, and targeting abilities.^[^ ^11^ ^]^ Thus, there is a burgeoning need for innovative and dependable strategies or medications to effectively manage oral diseases.

Since Gao et al. first reported that Fe_3_O_4_ NPs can generate ROS by exerting peroxidase (POD) activity to eradicate dental biofilms,^[^ ^12^ ^]^ the realm of ROS nanotherapy has made significant strides within the oral health sector, with a primary focus on the development of ROS‐related nanoplatforms and nanotherapeutic strategies.^[^ ^13^ ^]^ ROS‐based nanomaterials such as nanozymes, various nanodelivery platforms, and nanorobots,^[^ ^14^, ^15^, ^16^ ^]^ boast unique compositions and sophisticated designs that confer specific advantages for oral applications. These include size effects, surface modifiability, high surface area‐to‐volume ratios, and targeting capabilities, which largely surmount the limitations associated with traditional pharmaceuticals.^[^ ^17^ ^]^ Mechanistically, these novel ROS nano‐regulators can directly modulate the activity of redox metabolism‐related enzymes or regulate the activity factors in response to both endogenous (e.g., intracellular pH, metabolites) and exogenous (e.g., light, sound, magnetism), ultimately achieving precise control over ROS concentrations.^[^ ^16^, ^18^, ^19^, ^20^ ^]^ This approach offers a more potent and convenient alternative for the management of oral diseases.

To date, there has been no comprehensive and in‐depth elucidation of the mechanisms by which nanomaterials regulate intracellular ROS levels and their applications in oral diseases. Therefore, this paper reviews the generation and biology of ROS, as well as the impact of nanomaterials on ROS. It delves into the potential regulatory mechanisms of ROS‐based nanomaterials in guiding intracellular ROS evolution. Furthermore, it analyzes the current status and feasible strategies of these nanomaterials in the oral cavity from the perspectives of ROS clearance and generation (**Table** **1**). The aim is to inspire and guide researchers in the development of innovative nanomaterials for treating oral diseases.

**Table 1:** Summary of various ROS‐based nano‐platforms, analyzing their potential molecular mechanisms in the treatment of oral diseases, with particular emphasis on their prominent performance in application.

Classification Of ROS‐based nanoplatforms	Therapeutic nanoplatforms	Nanomaterials	Functional agents	Mechanisms	Performances	Efficacy	Applications	Ref.
**ROS‐Scavenging Nanoplatforms**	**Antioxidant Nanozymes**	CoO − Ir	CoOx, Ir	SOD, CAT	Providing cascade ROS scavenging propertity, and ultrafast antioxidase ‐like activities	Exerting anti‐inflammatory activity; Improving the differentiative capacity	Periodontitis	[165]
		Cerium oxide nanozyme	CeO_2_	SOD, CAT; Inhibiting the MAPK–NF κ B signaling pathway	/	Exerting anti‐inflammatory and antioxidant activity	Periodontitis	[194]
		TM/BHT/CuTA	CuTA NSs	SOD, CAT; Shifting microglial activation from M1 to M2 phenotype	Achieving responsive and on‐demand releasing; Increasing bioavailability; Achieving selectively accumulate in inflammation sites	Exerting anti‐inflammatory, antioxidant and antibacterial activity, and osteogenic functions	Periodontitis	[16]
		DM	MnO_2_	SOD, CAT	Showing remarkable efficiency, safety, and biocompatibility	Exerting anti‐inflammatory and antioxidant activity; Promoting reosseointegration	Peri‐Implantitis	[161]
		VB_2_‐IONzymes	iron oxide nanozymes, vitamin B_2_	POD, CAT, SOD	Improving the catalytic properties of IONzymes; Supplying VB_2_ and iron to avoid the deficiency of these nutrients in patients.	Exerting anti‐inflammatory and antibacterial activity	Mouth Ulcer	[162]
		Pt@PCN222‐Mn	Pt@PCN222‐Mn	SOD, CAT; Inhibiting the ROS‐NF κ B and MAPK signaling pathway;	Increasing water dispersibility	Exerting anti‐inflammatory andanti‐apoptotic effects to protect cartilage.	Temporomandibular joint osteoarthritis (TMJ OA)	[164]
		i‐PMMA	Ce NPs	Suppressing degradation of ECM and apoptosis of chondrocytes. SOD, CAT; Increasing expression of homeostasis‐related proteins, SOD and ERK and Akt, and cellular migration	Improving biocompatibility and resistance to microbial adhesion of multiple species; Cytoprotection	Exerting anti‐inflammatory and antioxidant activity	Maxillofacial defect	[195]
	**Antioxidant** **nanoplatform integrated with** **ROS Scavenger**	PGO‐PHA‐AG	PGO, PHA	Activating Ca^2+^ channels; Shifting microglial activation from M1 to M2 phenotype by mediating glycolytic and RhoA/ROCK pathways	Providing good cell conductive effect; Promoting the stable water dispersibility and cell adhesion; Improving cell immunomodulatory abilities; Reducing adverse reaction.	Synergistic effects of good conductive, ROS‐scavenging, anti‐inflammatory, and immunomodulatory abilities.	Periodontal bone defect in diabetes	[196]
		MSN@Ce@PEG	CeO_2_, MSNs	SOD, CAT; Releasing Si ions to promote PDLSCs osteogenesis differentiation	Improving dispersion and biocompatibility; Enhanced circulation time and permeability in tissues, and reduced hemolytic activity by modifying PEG on the surface of NPs.	Exerting anti‐inflammatory activity; Protecting cells from aging and improving the differentiative capacity of hPDLSCs	Periodontal bone defect	[95]
		Fe_3_O_4_‐CaO_2_	Fe_3_O_4_, CaO_2_	POD, CAT, SOD	Increasing bioavailability controlled drug release and self‐supplying substrate(H_2_O_2_)	Decreasing bacterial metabolic activity; Exerting antibacterial activity	Root canal biofilm infection	[156]
		PG‐CeO_2_	CeO_2_	SOD, CAT	Improving mechanical properties and biocompatibility	Improving the proliferation and differentiative capacity of hPDLSCs	Periodontal bone defect	[197]
		2D MOF (CuTCPP‐Fe_2_O_3_)	CuTCPP, Fe_2_O_3_	POD, CAT	Biocompatible and biodegradable; Slow ion release of CuTCPP‐Fe_2_O_3_ in the relatively long‐term; Promoting cell proliferation and angiogenesis	Exerting antibacterial and anti‐inflammatory activity;	Periodontitis	[198]
		PPTB NPs	BAPTA‐AM	“glut” Ca^2+^ around and inside mitochondria; Controlling the sustained opening of mPTPs	pH responsive property; Increasing mitochondria‐targeting;	Exerting anti‐inflammatory activity	Periodontitis	[117]
		PEG‐ss‐PCL NPs	N‐acetylcysteine (NAC)	Endogenous antioxidant GSH	ROS‐responsive properties; Increasing targeting, safty and efficiency of drug delivery	Exerting anti‐inflammatory activity; Improving the differentiative capacity of hPDLSCs	Periodontitis	[41]
		NAC‐CPDs	carbonized polymer dots(CPDs), N‐acetylcysteine (NAC)	Scavenging mitochondria ROS Suppressing the expression of TLR4 and NF‐κB, and activating Keap1/Nrf2	Increasing mitochondria‐targeting; Providing certain periodontal tissue enrichment ability	Exerting anti‐inflammatory and antioxidant activity; Promoting the differentiative capacity of hPDLSCs	Periodontitis	[199]
**ROS‐Generating Nanoplatforms**		MPB‐BA	mesoporous Prussian blue (MPB); Baicalein (BA)	POD, CAT, SOD; Shifting microglial activation from M1 to M2 phenotype by suppressing NF‐κB and upregulating Nrf2 and antioxidant gene	Promoting PTT properties and NIR‐controlled release of drug	Exerting anti‐inflammatory, antibacterial and antioxidant activity;	Periodontitis	[167]
		PDMO hydrogel	MnO_2_ NPs, DOPA	SOD, CAT	PTT properties; Providing the injectable and adhesive properties; Improving the medications concentration, retention time and biocompatibility.	Exerting antioxidant and antibacterial activity	Periodontitis	[81]
		MitoQ@PssL NPs	Mitoquinone, PssL NPs	Scavenging mitochondria ROS; Activating the PINK/Parkin signaling pathway to induce mitophagy	ROS responsive property; Achieving drug precise delivery and releasing; Low toxicity, high biocompatibility and stable physicochemical properties	Exerting anti‐inflammatory activity, and accelerating osteogenesis	Periodontitis	[115]
		HMPB@Cur@PDA	HMPB, Cur	POD, CAT, SOD; Shifting microglial activation from M1 to M2 phenotype	ROS and pH‐responsive properties; Increasing targeting, safty and efficiency of drug delivery	Exerting anti‐inflammatory and antioxidant activity;	Maxillofacial infection	[166]
		Met@TA‐ZIF‐PSG patch	PDA‐mSF, TA, Met‐ZIF	inhibition of M1 macrophages/activation of M2 macrophages activity	Promoting cell adhesion; Increasing targeting, sustained drug‐release ang concentration; Enhancing biocompatibility, biodegradability, and mechanical properties.	Exerting anti‐inflammatory, antioxidant, antiaging and immunomodulatory abilities; Improving periodontal ligament reconstruction;	Periodontal bone defect in diabetes	[79]
	**Nanozymes**	CAT‐NP	Fe_3_O_4_	POD	pH‐responsive property; High biocompatibility and ability to penetrate biological matrices	Exerting antibacterial activity; reducing apatite demineralization	Dental caries	[12]
		Dex‐NZM	Fe_3_O_4_	POD	pH‐responsive properties; Increasing targeting; Providing stability without blocking catalysis	Exerting antibacterial activity	Dental caries	[121]
		Dex‐IONP‐GOx	Fe_3_O_4,_ GOx	GOx, POD	pH‐responsive property; Increasing targeting and preferentially toward biofilms; Self‐supplying substrate(H_2_O_2_)	Exerting antibacterial activity	Dental caries	[42]
		Copper Doped Carbon Dots (Cu‐CDs)	Cu, CDs	POD, CAT	Increasing binding force with bacteria; Stimulating collagen deposition; Increasing bioavailability;	Exerting antibacterial activity; Against infection, accelerating healing and tooth nondestructive whitening	Oral Biofilm Infection, Wound Infection, Tooth Staining	[155]
		PPP‐MM‐S	SDF‐1, Metformin(Met)	mtETC Recruiting rBMSCs and reactivating the AMPK/β‐catenin pathway	Improving stepwise cargo release; Providing a novel therapeutic strategy of a bioinspired drugdelivery system emulating the natural bone healing cascade	Exerting antioxidant activity; Promoting the recruitment and differentiative capacity of rBMSCs	Periodontal bone defect in diabetes	[191]
		PAP34HB	ZnO NPs	Increasing cell membrane permeability, inhibit cellular respiratory enzymes and generate ROS	Increasing biocompatibility, safety, and longterm effectiveness; Better antibacterial and repair effects	Exerting antibacterial and anti‐inflammatory activity	Oral Soft Tissue Defect	[200]
	**Stimulus‐Responsive Nanoplatform**	BPDAH‐GPEGD	PDA/heparin (PDAH NPs), BMP‐2	/	Enhancing the compressive strength, modulus, toughness and antioxidant ability; Showing greater bone volume and enhancements in the quality and rate of bone regeneration	Exerting antioxidant activity; Promoting cell differentiation, mineralization deposits and mandibular bone regenerations.	Maxillofacial defect	[201]
		Oxygenated nanodiamonds (O‐NDs)	O‐NDs	POD	Increasing biocatalytic effects; Accelerating the wound healing around sites of periodontal infection in time.	Exerting antibacterial activity	Periodontal Bacterial Infection	[202]
		FDCDs	CDs	POD; Promoting macrophage recruitment	Promoting cytocompatibility and targeting; Increasing water dispersibility and stability	Exerting antibacterial activity	Persistent Endodontic Infections (PEIs)	[203]
		Janus piezotronic nanostructure	piezoPCL	Mitochondria ETC; Disturbing membrane stability, transmembrane transportation and carbon metabolism of bacteria.	Simplifying procedures of root canal reinfection treatment and polymer; Noninvasiveness of sonodynamic therapy; Preventing infection in the interspace between the implant and tissue	Exerting antibacterial activity; Preventing root canal reinfection	Implant‐associated infections (IAI)	[157]
		EI@Lipo	Evodiamine (EVO), indocyanine green (ICG)	POD	Synergistic efficiency of PDT and CDT; Improving water solubility of drug; Enhancing tumor passive targeting and sensitivity by promoting permeability and retention (EPR) effect	Exerting antitumor chemotherapeutic effect	Oral Squamous Cell Carcinoma, OSCC	[179]
		RC‐GMN	resveratrol (RES), photodynamic agent chlorine e6 (CE6)	Inhibiting tumor cellular oxygen consumption and reducing cellular ATP production; Elevating autophagic cell death	Promoting efficiency of PDT; Enhancing targeted accumulation in tumors and penetration and retention (EPR) effects	Exerting tumor killing effect	Oral Squamous Cell Carcinoma, OSCC	[178]
		PYT NP	Terbinafine; Photosensitizer, Y8	Reducing cholesterol; Increasing the production of ROS and damage‐associated molecular patterns	Providing photoimmunotherapy properties	Exerting anticancer immunity	Oral Squamous Cell Carcinoma, OSCC	[180]
		Ac‐DEVDD‐TPP	porphyrin nanofiber	Promoting apoptosis‐amplified assembly of porphyrin nanofibers and ^1^O_2_ generation by caspase‐3 cleavage and laser irradiation	Promoting efficiency of PDT; Providing an apoptosis‐amplified assembly strategy	Inducing tumour cell apoptosis and pyroptosis.	Oral Squamous Cell Carcinoma, OSCC	[182]
		CM@Co‐Fc@HCQ	Cobalt‐ferrocene MOF, hydroxychloroquine	Fenton reaction; Inhibiting the fusion of autophagy vesicles with lysosomes	Promoting homologous tumor targeting and biosafety; Increasing blood circulation of drug	Exerting tumor killing effect	Oral Squamous Cell Carcinoma, OSCC	[184]
		ICG‐rapamycin	indocyanine green (ICG), rapamycin	Elevating the ROS and temperature levels Shifting microglial activation from M1 to M2 phenotype	PTD and PTT properties; Increasing targeting; Improving the treatment efficiency and economic benefits; Realizing the treatment and prevention of both oral diseases	Exerting anti‐inflammatory and antibacterial activity	Oral Biofilm Infection	[19]
		TiO2@Ru@siRNA	Ru, TiO_2_ NPs	Reacting with water to produce ROS; Causing lysosomal damage, HIF‐1α gene silencing, and OSCC cell elimination	Increasing efficiency of PDT; Promoting tumor targeting, biosafety and biocompatibility	Hypoxia relief and pyroptosis induction; Exerting anticancer immunity	Oral Squamous Cell Carcinoma, OSCC	[181]
		ZFH	Fisetin (FS); Zein (Zn)	Reducing OSCC‐specific serum biomarkers levels, histologic tumor grade and increased caspase‐3 level	pH‐responsive drug release and dual targeting effect; Increasing accumulation of the loaded drug within the tumor tissue	Exerting tumor killing effect	Oral Squamous Cell Carcinoma, OSCC	[204]
	**Micro‐Nanorobots**	surface topography‐adaptive robotic superstructures (STARS)	Fe_3_O_4_(IONPs)	POD, CAT	Providing “toothbrushing‐like” and “flossing‐like” action with antibacterial activity in real‐time; Achieving mechanochemical removal and multikingdom pathogen detection.	Exerting antibacterial activity	Oral Biofilm Infection	[138]
		IONPs microrobots	Fe_3_O_4_	POD, CAT	Promoting precise spatial targeting; Increasing enhance localized delivery of NPs High binding affinity and adhesion with fungal	Exerting antibacterial activity	Fungal Infection	[140]
		catalytic antimicrobial robots (CARs)	Fe_3_O_4_	POD, CAT	Precisely and controllably kill, degrade and remove biofilms with remarkable efficiency; Addressing both the biological and mechanical traits associated with biofilm recalcitrance and reinfection	Exerting antibacterial activity	Oral Biofilm Infection	[147]
		black‐TiO_2_/Ag nanorobots	B‐TiO_2_, Ag	Enhanced release of ROS and Ag ions under light irradiation	Increasing targeting; Showing self‐propulsion activity and remotely controlled	Exerting autonomous movement and antibacterial activity	Oral Biofilm Infection	[15]

## 2 ROS: Generation and Biology

### 2.1 Generation of the ROS

ROS encompass a spectrum of chemically reactive oxygen‐containing molecules, ions, and free radicals, including superoxide anion (O_2_^•−^), hydrogen peroxide (H_2_O_2_), hydroxyl radical (OH•), and singlet oxygen (^1^O_2_),^[^ ^21^ ^]^ and different types of ROS can undergo interconversion. Extensive research indicates that ROS sources can be categorized into cellular and non‐cellular origins.

During normal cellular metabolism, the principal endogenous enzymatic sources of O_2_^•−^ and H_2_O_2_ are nicotinamide adenine dinucleotide phosphate (NADPH) oxidases (NOXs) and the mitochondrial electron transport chain (mtETC).^[^ ^22^, ^23^ ^]^ The NOX family, a transmembrane enzyme group consisting of seven members (NOX1‐5 and Duox1‐2), is localized at distinct sites on the cytoplasmic membrane, contributing to the site‐specific generation of ROS.^[^ ^22^, ^24^ ^]^ NOXs generate O_2_^•−^ by consuming NADPH, which then either spontaneously dismutates or further converts to H_2_O_2_ under the catalysis of superoxide dismutase (SOD). This is also an important mechanism by which NOXs kill microbes and deactivate microbial virulence factors.^[^ ^25^ ^]^ In the mtETC, complexes I‐IV release O_2_^•−^ and H_2_O_2_ (collectively termed mtROS) into the mitochondrial matrix, cristae, and intermembrane space through the interaction of electrons with oxygen.^[^ ^26^ ^]^ The topological differences in mtETC ROS production have functional significance, as they mediate different types of redox modifications on biomolecules in a time‐ and sequence‐specific manner, allowing for spatially localized redox reactions.^[^ ^27^ ^]^ It is worth noting that the generation of mtROS is closely related to Ca^2+^, which may be associated with the ROS‐induced ROS release (RIRR) effect of Ca^2+^ (where ROS produced by one mitochondrion induce other mitochondria to release ROS), thus maintaining mitochondrial homeostasis is crucial for ensuring beneficial ROS signaling within cells.^[^ ^28^ ^]^ Beyond NOXs and mtETC, ROS is also generated by various other oxidases in subcellular locations, including the endoplasmic reticulum (ER), peroxisomes, and SOD, which facilitate the exchange and transport of ROS from different sources, sustaining a stable and orderly redox regulatory network and keeping ROS levels within a normal threshold, thereby enhancing their efficacy.^[^ ^29^ ^]^

**Figure 1:**
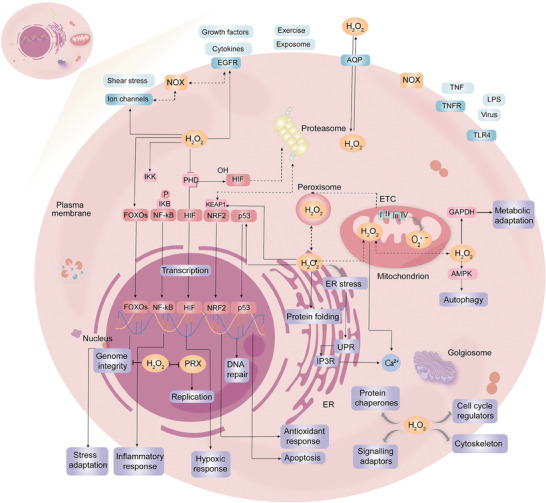
Pleiotropy of redox signaling in cell biology. Cells respond to a plethora of exogenous stimuli (pale yellow) via various receptors and channels (dark blue) in the plasma membrane, initiating the generation of O_2_·^−^ and H_2_O_2_ (orange), which subsequently act on a plethora of cellular targets (dark red), thereby eliciting diverse biological activities (purple). These activities are highly pleiotropic, including the regulation of stress adaptation (including antioxidant response), inflammatory response, cell death, and metabolic adaptation.

Apart from endogenous cellular sources, ROS is also generated by cumulative environmental exposures, such as drugs, toxins, and pollutants, as well as physical stressors (infrared, X‐rays, and other ionizing radiation).^[^ ^30^ ^]^ With the most typical being ^1^O_2_, many studies have clarified that ^1^O_2_ is typically produced through exogenous photochemical reactions rather than endogenous biological reactions.^[^ ^31^, ^32^ ^]^ Moreover, in nature, these ubiquitous ROS are synthesized through various mechanisms. For example, many nanomaterials can guide the generation of ROS through various catalytic reactions, facilitating the transformation of exogenous energy into endogenous chemical energy of ROS.^[^ ^20^, ^33^ ^]^ In conclusion, ROS has numerous sources, and understanding the subcellular localization or non‐cellular sources of these ROS species can provide clues for further exploring the biology of ROS, such as oxidative stress and subsequent pathological behaviors(**Figure** **1**).

### 2.2 Biology of the ROS

ROS are well‐documented as intracellular signaling molecules, playing a pivotal role in cellular communication.^[^ ^21^ ^]^ Their reactivity, specificity, and diffusibility position ROS as key mediators of intercellular dialogue, influencing their biochemical roles within physiological environments.^[^ ^1^, ^13^, ^21^ ^]^ For instance, the H_2_O_2_‐mediated signaling pathway involving cysteine (Cys) residue undergoes oxidative modifications altering protein activities, thereby participating in the regulation of various physiological activities such as cell proliferation, differentiation, migration, and apoptosis.^[^ ^34^ ^]^ It is noteworthy that ROS, as the primary mediators of redox signaling, exhibit a “double‐edged sword” effect, while low to moderate concentrations of ROS can act as signaling molecules, excessive ROS can overwhelm cellular antioxidant defenses, leading to cell death.^[^ ^35^ ^]^

Increasing evidence suggests a close association between ROS and organismal function and tissue integrity. At the cellular level, different concentrations and types of ROS exert varying effects on cells and tissues, with the same concentration of ROS displaying different effects on different cells and tissues.^[^ ^36^, ^37^ ^]^ For example, low levels of ROS participate in multiple cell signaling pathways by modulating the activity of oxidative targets, including cellular signaling proteins (FOXOs, NF‐κB, Keap1‐Nrf2‐ARE, and HIF‐ɑ), ion channels, and transport proteins (Ca^2+^ and mPTP), as well as modifying protein kinases and the ubiquitin‐proteasome system, playing crucial roles in pathogen defense, oxygen regulation, and induction of apoptosis.^[^ ^2^, ^38^ ^]^ Conversely, high concentrations of ROS can induce oxidative stress, a detrimental process that causes severe oxidative damage to cellular structures.^[^ ^39^ ^]^ For instance, during periodontal tissue regeneration, unresolved local infections and inflammation lead to excessive ROS production, resulting in lipid, protein, and DNA peroxidation, directly impairing cells and tissues.^[^ ^40^ ^]^ Additionally, ROS‐induced inflammatory environments inhibit bone formation markers while inducing an increase in osteoclastogenesis, disrupting the homeostasis between bone formation and resorption, exacerbating periodontal inflammatory responses and tissue destruction.^[^ ^41^ ^]^ However, these overexpressed ROS can also aid the host in combating microbes by triggering the degradation of bacterial extracellular polymeric substances (EPS) to induce pathogen death, which is of significant importance for controlling dental plaque biofilms.^[^ ^42^ ^]^

ROS biology is a highly complex field, where fluctuations in ROS concentrations largely dictate the fate of organisms. Its involvement in redox signaling cascades affects the functions of macromolecules such as proteins, potentially leading to changes in signal transduction, enzyme activities, gene transcription, and the integrity of membranes and genomes, thereby impacting the life activities of organisms.^[^ ^1^, ^43^ ^]^ Therefore, the strict regulation of ROS concentrations is essential for guiding and maintaining the normal functions of cells and tissues, and for promoting organismal health.

## 3 ROS‐Scavenging Nanoplatforms

**Figure 2:**
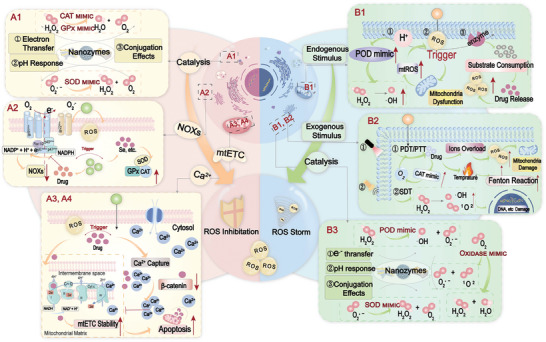
Nanomaterials affect ROS scavenging and generation. **(A1)** Catalytic elimination of ROS by antioxidant nanozymes. **(A2)** Nanosystems regulating endogenous enzymes associated with ROS generation and scavenging. **(A3,A4)** Regulation of ROS generation via mitochondrial electron transport chain(mtETC) and Ca^2+^ channels by nanosystems. **(B1)** Nanoplatforms mediating ROS generation through endogenous response (pH, ROS, Enzymes, etc.). **(B2)** Nanoplatforms mediating ROS generation through exogenous response (Light, Heat, Sound, etc.). **(B3)** ROS generation catalyzed by nanozymes.

The recent advancements in ROS nanotherapies have been significantly bolstered by the development of ROS‐scavenging nanomaterials for antioxidant therapy. Given the diverse and intricate nature of diseases such as inflammation, these nanomaterials can be meticulously customized to align with specific biological contexts, including location, physicochemical cues, and immune environment. Enhancing their biostability during transport and their accumulation at pathological sites maximizes their therapeutic efficacy. Based on their ROS‐scavenging mechanisms, nanomaterials are categorized into two primary groups: i) antioxidant nanozymes, which catalyze the removal of ROS; and ii) non‐enzymatic antioxidant nanoplatforms, which deliver upstream regulatory compounds that target ROS generation or transport antioxidant drugs This classification underscores the strategic approach to harnessing nanomaterials for precise and effective ROS management in therapeutic applications. (**Figure** **2**).

## 4 Antioxidant Nanozymes

Since the unveiling of an artificial POD enzyme based on ferromagnetic magnetite nanoparticles (Fe_3_O_4_ NPs) by Gao et al. in 2007,^[^ ^44^ ^]^ enzyme‐based nanomaterial science has witnessed substantial advancements, primarily manifested in the development and application of novel nanozymes.^[^ ^45^, ^46^, ^47^ ^]^ Nanozymes possess a dual identity of both enzymes and nanomaterials, endowed with the capability to catalyze substrate conversion akin to natural enzymes.^[^ ^48^ ^]^ Leveraging the advantages of nanodesign and application, they greatly overcome issues such as low stability and poor bioavailability inherent in natural enzymes.^[^ ^49^ ^]^ Currently developed antioxidant nanozymes mainly include metal‐based nanozymes (MNPs), carbon‐based nanozymes (CNMs), polymeric nanozymes (PMNs) and inorganic‐organic hybrid nanozymes (IOHNs).and At the molecular level, their catalytic activity primarily revolves around redox reactions, with enzyme activities related to ROS scavenging including catalase (CAT), SOD, and glutathione peroxidase (GPx).^[^ ^50^ ^]^ Despite their similar catalytic activities, these nanozymes typically exhibit different catalytic mechanisms. In the following sections, we will explore different types of antioxidant nanozymes capable of clearing intracellular ROS by analyzing their distinct catalytic mechanisms, including electron transfer, pH responsiveness, and conjugation effects.

### 4.1 Metal‐Based Nanozymes, MNPs

MNPs are currently the most widely used in antioxidant therapy, mainly including cerium (Ce), manganese (Mn), cobalt (Co), platinum (Pt), iron (Fe), and copper (Cu) NPs.^[^ ^51^ ^]^ Their catalytic mechanism mainly involves electron transfer between metal oxidation states. Multivalent metal ions (M) serve as electron donors, utilizing substrates such as O_2_ or H_2_O_2_ as electron acceptors, leading to the switching between M^(n)+^ and M^(n+1)+^, thus completing the catalytic cycle.^[^ ^52^ ^]^

**Figure 3:**
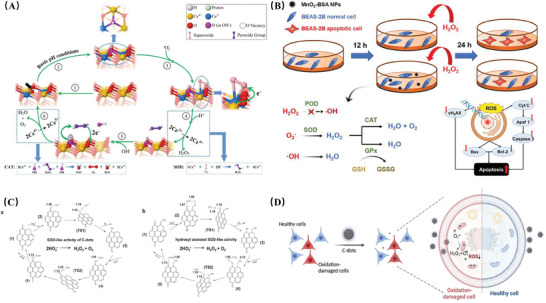
A) Schematic illustration showing changes in surface chemistry of CeO_2_ driven by SOD and CAT biomimetic reactions under basic conditions; black arrows indicate the locations of oxygen vacancies; and the surface state will transfer directly from step (5) to step (2) if both OH– ions are supplied by an aqueous environment. Adapted with permission.^[^ ^54^ ^]^ Copyright 2022, American Chemical Society. B) Schematic illustration of the MnO_2_ nanoparticles (MnO_2_−BSA NPs) coated with BSA. Adapted with permission.^[^ ^57^ ^]^ Copyright 2022, American Chemical Society. C) Proposed SOD‐like activity of C‐dot nanozyme with and without hydroxyl groups. a. Proposed reaction pathway to achieve the SOD‐like catalytic cycle of C‐dot nanozyme without hydroxyl groups. b. Proposed reaction pathway to achieve the SOD‐like catalytic cycle of C‐dot nanozyme with hydroxyl groups. D) Illustration of the selective targeting and ROS scavenging ability of C‐dot SOD nanozymes to oxidation‐damaged cells. Adapted with permission.^[^ ^71^ ^]^ Copyright 2023, Springer Nature.

For example, Ce‐based nanozymes exhibit SOD and CAT mimetic activities, attributed to the reversible Ce^3+^/Ce^4+^ redox electron pairs and oxygen vacancies of Ce, where the proportion of Ce^3+^/Ce^4+^ determines the catalytic activity of CeO_2_ NPs(**Figure** **3**A).^[^ ^53^, ^54^ ^]^ In comparison to Ce‐based nanozymes, Mn‐based nanozymes inherently possess multiple oxidation states, providing stronger and more diverse antioxidant enzyme catalytic activities. For instance, Mn_3_O_4_ nanozymes, due to the presence of Mn^2+^ and Mn^3+^ in the bivalent and trivalent oxidation states, exhibit significant SOD and CAT‐like activities and demonstrate better thermal stability than natural SOD and CAT, showing a 1.5‐fold enhancement in antioxidant activity compared to CeO_2_ NPs at the same dosage.^[^ ^55^ ^]^ Therefore, the excellent ROS scavenging capability of Mn‐based nanozymes offers promising prospects in antioxidant therapy.

It is worth noting that the activity of MNPs is not only regulated by their own structure and composition but also influenced by physical properties such as temperature, surface modification, and light exposure. These physical properties determine the number of catalytic centers, nanocrystal facets, and electron transfer efficiency.^[^ ^50^, ^56^ ^]^ For example, MnO_2_ NPs modified with bovine serum albumin (BSA) (MnO_2_‐BSA) not only increase the types of enzyme catalytic activities (SOD, CAT, and GPx) but also enhance the overall catalytic process efficiency. While exhibiting SOD‐like activity to eliminate ROS, they achieve self‐supply of substrate H_2_O_2_, enabling deeper utilization of products(Figure 3B).^[^ ^57^ ^]^

In contrast to the above, besides electron transfer, the catalytic activity of Fe is pH‐dependent. Different pH levels affect the timing of H^+^ and OH^−^ adsorption on the surface of Fe‐based nanozymes, determining various pathways for H_2_O_2_ decomposition. Thus, Fe‐based nanozymes can function as both antioxidants (CAT‐like activity) and oxidants (POD‐like activity). Gu et al. confirmed this by finding that Fe_3_O_4_ NPs (IONPs) catalyze the production of OH• from H_2_O_2_ under acidic conditions (pH ≤ 4) in lysosomes. At neutral pH, the increased precipitation of Fe(OH)_3_ leads to a decrease in Fe^3+^ concentration, directly reducing the rate of the Fenton reaction, wherein IONPs directly catalyze the production of H_2_O and O_2_ from H_2_O_2_.^[^ ^58^ ^]^ Unlike Fe, although Cu‐based nanozymes also possess this property, they exhibit three‐enzyme‐like activities (POD, GPx, and CAT) in response to pH changes. They have recently been used as antibacterial agents and antioxidants for combined therapy in infections and injuries, showing excellent efficacy.^[^ ^59^ ^]^ Other metal nanoparticles and their oxides, such as Au, Pt, and Pd, have also been found to exhibit pH‐dependent enzyme‐like activities.^[^ ^60^, ^61^, ^62^ ^]^

To some extent, the discovery of this property provides more possibilities for the design of MNPs, showing advantages in combined antibacterial and antioxidant therapy. However, in practical applications, due to the inability to accurately predict intracellular pH changes and other possible interfering factors, the application and timing selection of such nanozymes in some mild oxidative stress‐related diseases need further consideration to avoid adverse therapeutic effects. Meanwhile, for complex diseases where inducing bactericidal or anticancer effects primarily through mediated ROS elevation is required, nanozymes can be prioritized under the premise of ensuring non‐toxic safety.

### 4.2 Carbon‐Based Nanozymes, CNMs

The catalytic activity of CNMs and their hybrids has been widely reported in recent years, including fullerene and its derivatives, graphene oxide (GO), graphene quantum dots (GOQDs), carbon dots (CDs), graphdiyne (GDY), and carbon quantum dots (CQDs).^[^ ^63^ ^]^ As a new type of nanozyme, CNMs possess unique catalytic mechanisms in ROS scavenging, primarily attributed to their abundant functional groups (such as amino, hydroxyl, and carboxyl groups) modification and the conjugated electron transfer capability of π–π systems.^[^ ^63^, ^64^ ^]^

Initially, DUANG et al. elucidated the catalytic mechanism of SOD‐like activity of fullerene C60 derivatives (C3), which involves electrostatic attraction to transfer O_2_^•−^ to the electron‐deficient regions on the surface, subsequent stabilization of O_2_^•−^ by carboxyl groups on the C3 derivative surface through proton hydrogen bonds, and final removal of O_2_^•−^ through dismutation.^[^ ^65^ ^]^ GO nanozymes, as another type of carbon‐based ROS scavenger, unlike fullerene, their radical scavenging capability is not dependent on oxygen‐containing functional groups but closely related to their inherent sp2 hybridized carbon network,^[^ ^66^ ^]^ which endows GO‐based nanozymes with stronger conjugated electron transfer effects, making them highly promising radical scavengers. Moreover, various strategies can enhance the catalytic activity of GO, such as doping with different elements,^[^ ^67^ ^]^ altering the size,^[^ ^68^ ^]^ or constructing GO‐based hybrid nanozymes with synergistic effects.^[^ ^69^, ^70^ ^]^ It is noteworthy that CDs nanozymes have garnered considerable attention due to their high catalytic activity (comparable to natural enzymes) and facile surface functionalization. In addition to sharing similar catalytic mechanisms and activity enhancement strategies with GO,^[^ ^71^ ^]^ in a representative work, CDs nanozymes with SOD‐like activity were found to selectively target oxidative damage in cells and mitochondria, coupled with their high catalytic activity, providing advantages in clearing intracellular ROS.^[^ ^72^ ^]^ Other types of CNMs such as GDY and carbon nanotubes exhibit similar antioxidant catalytic activity and mechanisms, which are not elaborated here(Figure 3C,D).^[^ ^73^ ^]^

Indeed, compared to MNPs, carbon, as the most abundant element in nature, may serve as a more economical and environmentally friendly basic building block for nanozyme synthesis. Furthermore, the unique electronic structure, geometric structure, and abundant functional groups of CNMs can effectively mimic the catalytic centers of natural enzymes, enhancing their accessibility. Based on the outstanding antioxidant activity of CNMs, numerous effective carbon‐based nanotherapeutic agents or treatment methods have been developed to date, demonstrating significant clinical efficacy, which will be elaborated on in detail in the application section.

### 4.3 Polymeric Nanozymes, PMNs

Polymeric nanozymes (PMNs) represent a novel class of artificial enzymes that have emerged within the realm of polymeric nanoparticles in catalysis. Over recent years, these nanomaterials have garnered significant advancements in the fields of materials science and bioscience. Characterized by their high catalytic activity, tunability, mild reaction conditions, robust stability, and unique multi‐enzymatic activities, PMNs offer a distinct advantage over traditional enzymes in that they can be more readily tailored and engineered to meet specific application requirements.^[^ ^74^ ^]^ Research into polymeric nanozymes encompasses a diverse array of raw materials, including metal‐based, metal‐free, and metal‐organic framework‐based substrates, each exhibiting varying degrees of enzyme‐like catalytic activity.^[^ ^75^ ^]^

**Figure 4:**
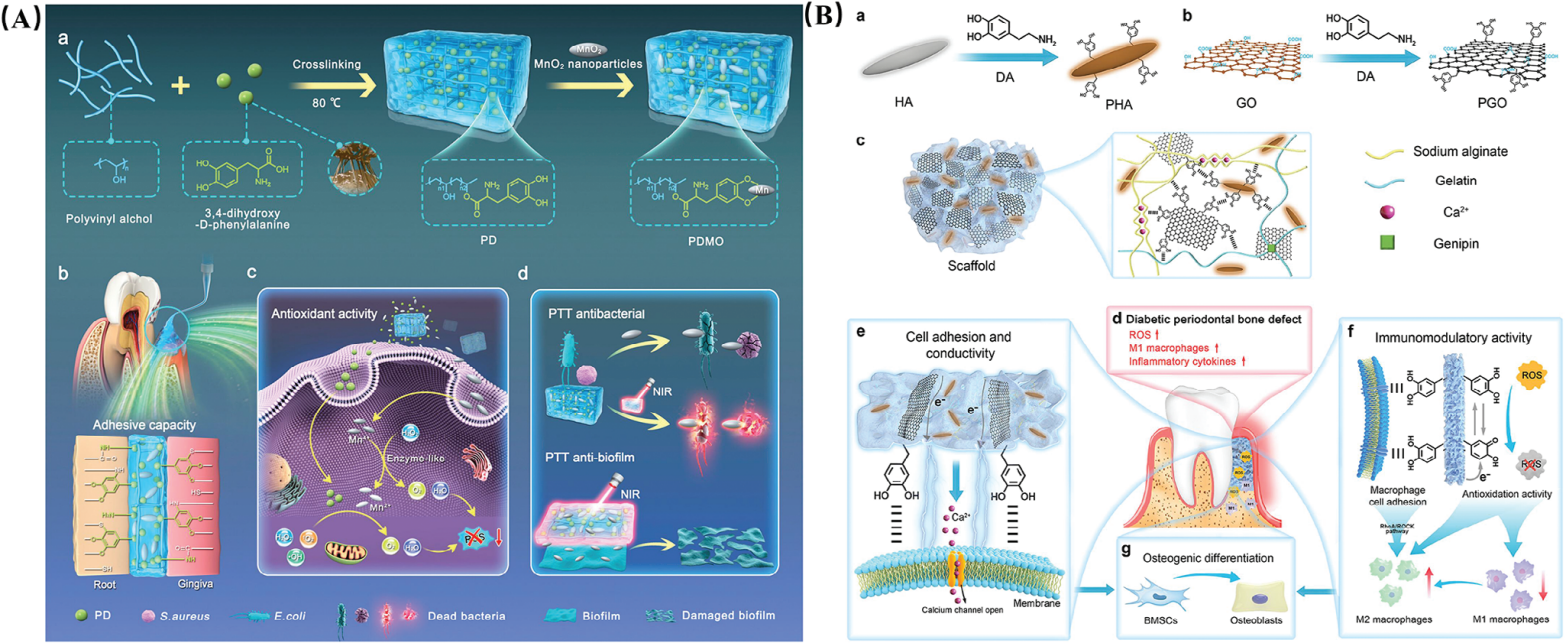
A) Schematic illustration of the synthesis of the PGO‐PHA‐AG scaffold with multifunctional properties for potential application in periodontal bone regeneration in diabetes. Adapted with permission.^[^ ^79^ ^]^ Copyright.2022, Elsevier. B) Schematic illustration of the PDMO hydrogel for treatment of periodontitis. Adapted with permission.^[^ ^81^ ^]^ Copyright 2023, American Chemical Society.

Polydopamine nanoparticles (PDA NPs) represent the quintessential class of antioxidant polymeric nanozymes, a biomimetic material inspired by the adhesive proteins of mussels.^[^ ^76^ ^]^ These nanoparticles possess an abundance of reductive functional groups, such as catechols and imines, which endow them with a unique multifaceted antioxidant capacity against various ROS. This includes direct scavenging,^[^ ^77^ ^]^ promotion of autophagy, ROS‐responsive drug release,^[^ ^78^ ^]^ and modulation of endogenous antioxidant systems. The synergistic action of these mechanisms renders PDA NPs an effective antioxidant nanomaterial. For instance, due to their ability to directly quench a variety of free radicals in inflammatory microenvironments, PDA NPs are currently being utilized as highly efficient ROS scavengers in numerous antioxidant therapy studies.^[^ ^77^ ^]^ In a seminal work, Lu et al. integrated PDA‐modified GO and hydroxyapatite nanoparticles (HA) into an alginate/gelatin (AG) synthetic conductive scaffold (**Figure** **4**A). The resulting gelatin scaffold facilitated the transmission of endogenous electrical signals to cells, activating Ca^2+^ channels and modulating the activation state of macrophages to ameliorate the diabetic periodontal inflammatory microenvironment. This approach aimed to eliminate excessive ROS and enhance cell adhesion and alveolar bone regeneration, ultimately promoting the repair of periodontal bone in diabetes. This material shows exceptional potential in the immunomodulatory treatment of diabetic periodontitis.^[^ ^79^ ^]^ It is the excellent biocompatibility and antioxidant activity of these unique characteristics that have garnered widespread attention for PDA‐based nanomaterials in the realms of inflammation therapy, tissue repair, and the fabrication of biomimetic materials.

It is noteworthy that PMNs can act as a “bridge” in reactions, allowing for the introduction of other functional groups on their surfaces. This capability enables surface modification of a variety of materials, including metal oxides, precious metals, carbon materials, organic polymers, and mesoporous silica. Consequently, PMNs are not only capable of being generated within different nanostructures but also of more effectively targeting tissues, increasing selective accumulation of themselves and drugs without affecting healthy cells. Recent studies have further unveiled the potential of PMNs, particularly in the field of molecular catalyst immobilization. By employing immobilization techniques to anchor molecular catalysts (such as natural or artificial enzymes) onto polymeric nanocarriers, not only is the recovery and reuse of catalysts facilitated, but the stability, synergy, and catalytic efficiency of the materials are also enhanced.^[^ ^80^ ^]^ For instance, polyvinyl alcohol (PVA) is employed as a cross‐linking agent to complex mussel‐derived protein, specifically 3,4‐dihydroxy‐d‐phenylalanine (DOPA), with MnO_2_ NPs to form an adhesive hydrogel, referred to as PDMO (Figure 4B). This composite is being investigated for its antioxidant effects in periodontal disease. The hydrogel, enriched with catechol groups and MnO_2_ NPs, exhibits SOD like and CAT like activities, enabling an autocatalytic cascade that neutralizes excess ROS and O_2_, thereby alleviating hypoxia in the periodontal inflammatory microenvironment.^[^ ^81^ ^]^

Despite these promising results, numerous challenges remain to be addressed. The intricate synthesis of PMNs and their intermolecular interactions obscure the mechanisms underlying their antioxidant properties. Limited by current technological capabilities, the modulation of their antioxidant performance can only be achieved by adjusting certain parameters during synthesis. Consequently, the regulation of PMNs' antioxidant capabilities is still in its infancy, necessitating the development of more effective strategies to augment the antioxidant activity of PMNs.

### 4.4 Inorganic–Organic Hybrid Nanozymes, IOHNs

Distinguished from the aforementioned inorganic or organic nanozymes, inorganic–organic hybrid nanozymes integrate organic molecules with inorganic nanoparticles, thereby inheriting the high stability and catalytic activity of inorganic materials while also incorporating the functionality and selectivity of organic molecules.^[^ ^82^ ^]^ The design and fabrication of these hybrid nanozymes typically involve the assembly of inorganic components such as metal oxides, metal nanoparticles, or carbon‐based materials with organic ligands or polymers through physical or chemical methods, resulting in composite materials with synergistic effects.^[^ ^82^ ^]^

**Figure 5:**
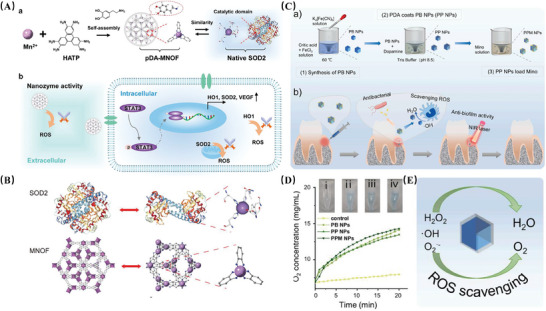
A) Schematics of an SOD2‐inspired manganese‐organic framework to effectively scavenge ROS. a) Schematics of fabrication of pDA‐MNOF that possessed a bioinspired structure mimicking the catalytic domain of native SOD2.b) Schematics of pDA‐MNOF scavenges extracellular ROS with nanozyme activities, while reducing intracellular ROS through upregulating endogenous antioxidant enzymes, HO1and SOD2 via activating STAT3 signaling. B) Structure of native SOD2 enzyme and MNOF. Purple balls represent manganese atoms. Adapted with permission.^[^ ^88^ ^]^ Copyright 2023, John Wiley and Sons. C) Schematic illustrating of a) the step‐by‐step preparation process of the designed PPM NPs and b) the working mechanisms for periodontitis therapy. D) The catalase (CAT)‐like activity. Dissolved O_2_ concentration was measured by the dissolved oxygen meter. E) Schematic illustration of the ROS‐scavenging mechanisms of PP NPs through catalytic conversion of harmful ROS (OH•, O_2_⋅^−^, and H_2_O_2_) into non‐toxic molecules (H_2_O and O_2_). In vitro anti‐oxidation using PP NPs. Adapted with permission.^[^ ^97^ ^]^ Copyright.2023, Elsevier.

Metal–organic frameworks (MOFs), also known as porous coordination polymers (PCPs), stand out in the realm of inorganic–organic hybrid nanozyme research. These materials are formed through the self‐assembly of metal ions and organic ligands via coordination bonds, resulting in an infinite network structure that is characteristic of inorganic‐organic hybrid materials.^[^ ^83^ ^]^ As a class of porous crystalline materials, MOFs possess an exceptionally high surface area, porosity, and tunable pore size/functionalities, which render them highly promising for a broad range of applications in the fields of biocatalysis and medical engineering.^[^ ^83^, ^84^ ^]^ Based on their chemical properties, MOF‐based nanozymes can be categorized as follows: 1. Pristine MOFs. For instance, Prussian blue nanoparticles (PBNPs) and cobalt oxide nanoparticles (Co_3_O_4_ NPs) are capable of catalyzing the oxidation of substrates by H_2_O_2_ through direct electron transfer, demonstrating potent SOD and CAT‐like activities. Additionally, MOF/enzyme composite materials and MOF‐818 nanozymes, which possess SOD‐like and/or CAT‐like activities, have shown promise in antioxidant therapeutics.^[^ ^85^, ^86^ ^]^ 2. MOF‐based composite materials. This category includes MOF/Fe_3_O_4_, MOF/Au, and MOF/enzyme composites. By integrating MOFs with other materials, novel composites with synergistic effects are formed. These composites not only retain the inherent properties of MOFs but also introduce new functionalities. However, the susceptibility of pristine MOFs and MOF‐based composites to degradation under physiological conditions and their limited solubility in certain solvents significantly impede their practical applications.^[^ ^87^ ^]^ Consequently, they are often utilized as precursors or sacrificial templates in current practices. 3. MOF‐derived nanozymes. The introduction of guest molecules with catalytically active sites within the pore space of MOFs represents a significant advancement. As mentioned previously, MNPs, CNMs, and PMNs, due to their pivotal role in catalysis, are considered highly promising guest materials. For instance, the incorporation of manganese elements into pristine MOFs can yield novel MOF‐based nanozymes (MNOFs) that mimic the natural SOD2 catalytic structural domain, not only exhibiting SOD‐like activity but also upregulating the expression of endogenous antioxidant enzyme genes, thereby compensating for the inability of nanozymes to scavenge intracellular ROS(**Figure** **5**A,B).^[^ ^88^ ^]^ Furthermore, the introduction of active sites renders the design of MOF nanozymes more flexible and diverse, enabling control over the size, shape, and dispersion of MNPs and CNMs to achieve high stability and activity. It also effectively integrates their respective multiple advantages, significantly enhancing catalytic performance. For example, through ligand engineering strategies that adjust the atomic coordination positions and numbers, the spatial configuration and electronic structure of the materials can be altered to affect metal active centers, thereby enhancing catalytic performance or conferring new enzyme‐like activities, which are better suited for the treatment of diseases related to oxidative stress.^[^ ^89^, ^90^ ^]^

It is worth noting that in OIHNs, the highly porous structure provided by inorganic nanoparticles and the diverse functional groups supplied by the organic component account for the enhanced drug loading capacity and controlled release patterns, offering a multifunctional platform for drug delivery with improved characteristics.^[^ ^91^ ^]^ OIHNs can be broadly classified into two major categories based on their primary matrix components. The first category encompasses hybrid nanozymes based on inorganic nanoparticles, which primarily include silica, superparamagnetic iron oxide, ZnO, and hydroxyapatite, among others.^[^ ^91^ ^]^ Hybrid nanozymes grounded in inorganic nanoparticles are better positioned to overcome drawbacks such as low biocompatibility, non‐biodegradability, and rapid drug release.^[^ ^92^ ^]^ Among them, mesoporous and non‐porous silica nanoparticles are common types of inorganic nanoparticles. Due to their adjustable pore structures, large surface areas, ease of surface functionalization, and drug loading capabilities, there have been numerous reports on the encapsulation of organic components within silica matrices and the surface modification of groups present on the inner and outer surfaces with other organic molecules.^[^ ^93^, ^94^ ^]^ A quintessential example is the synthesis of a simple multifunctional inorganic‐organic hybrid nanozyme, MSN@Ce@PEG. Xu's team initially loaded CeO_2_ onto mesoporous silica (MSN), followed by modification with polyethylene glycol (PEG) to generate an organic nanohybrid of MSN. The results indicated that PEG modification enhanced the dispersibility and biocompatibility of MSN nanoparticles, while the incorporation of CeO_2_ endowed them with SOD and CAT‐like enzymatic activities. This effectively modulated the intracellular ROS, protected human periodontal ligament stem cells (hPDLSCs) from senescence, and enhanced their differentiation capacity, thereby preventing inflammatory destruction of periodontal tissues.^[^ ^95^ ^]^ The second category comprises hybrid nanozymes based on organic nanoparticles, such as carbon nanotubes, polydopamine, liposomes, micelles, and dendrimers.^[^ ^82^ ^]^ Unlike the first category, these organic nanoparticle‐based hybrid nanozymes possess more stable physicochemical properties, high permeability, and selectivity, which are a result of the unique physical structures of these organic nanoparticles. For instance, carbon nanotubes (CNTs), with their higher aspect ratios, appropriate dimensions, increased surface areas, ease of functionalization, and exceptional cellular penetration capabilities, have been widely utilized as drug carriers.^[^ ^96^ ^]^ PDA, mentioned previously, is a preferred choice in the fabrication of multifunctional composite nanomaterials due to its rich array of functional groups. In a study, the authors coated monodisperse Prussian blue nanoparticles (PB NPs) with PDA through the self‐polymerization of dopamine (PP NPs), followed by the loading of minocycline to prepare a multifunctional composite nanoplatform (PPM NPs). The nanoplatform based on PB NPs, facilely modified with PDA, not only significantly enhanced antibacterial effects through a combined therapeutic approach but also effectively scavenged cellular ROS using the enzymatic‐like activity of the nanozyme, contributing to the treatment of periodontitis(Figure 5C–E).^[^ ^97^ ^]^

In general, hybrid nanomaterials are engineered to overcome the shortcomings of individual systems. The type, composition, and interplay between inorganic and organic nanomaterials dictate the ultimate functionality of the hybrid. The inorganic component enhances biocompatibility, biodegradability, and targeted delivery capabilities, while the organic component provides additional attributes such as targeting, permeability, and optical activity. Moreover, due to their synergistic effects, new properties can emerge. It is these pronounced advantages that have led to their widespread documentation in research areas such as bone tissue engineering, antimicrobial, and anti‐inflammatory applications.

#### 4.4.1 Antioxidant Nanoplatform Combined with ROS Scavengers

Introduction of exogenous small molecule ROS scavengers is a common strategy to maintain and restore the redox homeostasis within cells.^[^ ^98^, ^99^, ^100^ ^]^ Notably, these ROS scavengers encompass antioxidant nanozymes, inflammation modulators, and other ROS‐clearing drugs. However, the complex multi‐layered biological barriers in vivo and inherent deficiencies of certain ROS scavengers hinder the efficacy of self‐recovery.^[^ ^101^ ^]^ Considering the advantages of nanomaterials such as small size, strong penetration, and high loading capacity, employing them as delivery vehicles may further enhance the bioavailability of small molecule antioxidants, achieving high‐targeted and precise release of drugs in cells and tissues.^[^ ^101^ ^]^ Based on their different roles in regulating ROS signaling nodes, integrated antioxidant nanoplatforms are categorized into three classes: i) enzyme‐regulating antioxidant nanosystems; ii) mitochondrial electron transport chain (mtETC) antioxidant nanosystems; iii) Ca^2+^ channel‐regulating nanosystems.

#### 4.4.2 Enzyme‐Regulating Antioxidant Nanosystem

The redox homeostasis of cells is maintained by ROS‐generating enzymes (such as NOXs) and ROS‐clearing enzymes (such as SOD, GPx). Once the balance is disrupted, excessive ROS become difficult to eliminate through endogenous antioxidant systems.^[^ ^102^, ^103^ ^]^ Therefore, utilizing NPs to regulate ROS‐related enzyme activities for treating oxidative stress‐related diseases is a promising strategy.

**Figure 6:**
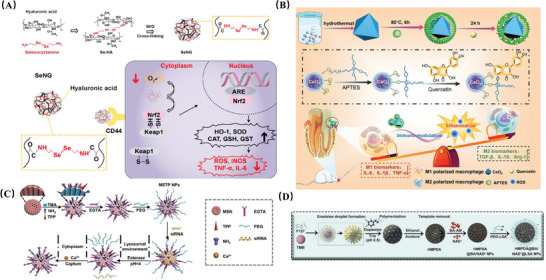
A) Schematic illustration of the protective effects of SeNG in a colitis model. This was achieved through the intrinsic anti‐ROS effect, coupled withtheCD44‐mediated targeting at the inflammatory site. Adapted with permission.^[^ ^107^ ^]^ Copyright 2022, American Chemical Society. B) Schematic illustration of CeO_2_@QU nanoparticles in the principle of synthesis and therapeutic mechanism. The chemical bonding of quercetin to functionalized CeO_2_ mainly through the acidic hydroxyl groups of quercetin breaking the Si‐O bond provided by APTES and then replacing the ethoxyl group. The anti‐inflammation activity of CeO_2_@QU relies on scavenging ROS and modulating the polarization of macrophages for the therapy of periodontitis. Adapted with permission.^[^ ^114^ ^]^ Copyright 2021, John Wiley and Sons. C) Schematic representation of the structure and function of METP NPs as well as how siβ‐catenin loaded METP NPs (METP/siβ‐catenin) sweep dysfunctional mitochondria and restore the function of mitochondria and MSCs. Adapted with permission.^[^ ^119^ ^]^ Copyright 2022, John Wiley and Sons. D) Schematic diagram of preparation of HMPDA@BA/NAD+@LSA NPs. Adapted with permission.^[^ ^120^ ^]^ Copyright 2023, John Wiley and Sons.

Some typical polymer NPs, such as polyoxalate‐based NPs, liposomes, and polysulfide and thiol‐ene‐based NPs, as well as PLAG, can undergo rapid hydrolysis in response to high ROS concentrations after chemical modification, thus achieving drug delivery.^[^ ^41^, ^104^ ^]^ Inspired by this, scholars have prepared NOXs nanoblockers HPOX and PVAX, which, upon hydrolysis of their H_2_O_2_‐sensitive polyoxalate main chains, immediately release loaded ROS scavengers (hydroxybenzyl alcohol (HBA) or vanillyl alcohol (VA)), subsequently reducing the expression of NOX2 and NOX4 in cells, thereby reducing the sources of ROS at the source.^[^ ^105^ ^]^ In addition to blocking NOXs, antioxidant nanosystems enhancing the expression of ROS‐clearing enzymes can be designed from the composition of endogenous antioxidant systems. For example, selenium (Se), a key and essential trace element in the GPx catalytic center to maintain the redox balance in the cell.^[^ ^106^ ^]^ Moreover, compared with inorganic and organic forms of Se, currently developed Se‐based nano systems, such as antioxidant nano systems Se@BSA NP, SeO_2_ porous NPs, oral nano gels (SeNG) (**Figure** **6**A), not only have higher biocompatibility and lower toxicity, but also directly increase the expression of endogenous antioxidant enzymes (SOD, CAT, glutathione‐S‐transferase (GST), and GPx‐1), thereby enhancing the protective effect of endogenous antioxidant systems, showing satisfactory efficacy in alleviating inflammation and tissue damage.^[^ ^107^, ^108^, ^109^ ^]^ This nano material, which regulates ROS‐clearing enzymes, not only overcomes the shortcomings of existing nano‐enzyme particles (such as low uptake rates, tissue barriers, etc.), but also provides a strategy to enhance the active clearance of ROS by endogenous antioxidant systems, offering a potential direction for finding new antioxidants to alleviate oxidative stress damage.

#### 4.4.3 mtETC Antioxidant Nanosystems

The mitochondrial electron transport chain (mtETC) serves as the primary site for ROS generation. When the mitochondrial system's homeostatic capacity is inadequate or defective, abnormal reactions induced by mitochondria can be pathogenic.^[^ ^110^ ^]^ Additionally, ROS itself is one of the mitochondrial damage‐associated molecular patterns (mtDAMPs), leading to dysfunction and reduced activity of mitochondrial components such as mtETC complexes and coenzyme Q, thereby exacerbating cellular oxidative damage.^[^ ^111^, ^112^ ^]^ Therefore, targeting mitochondria with specific antioxidant regulators may represent an effective approach to restore mtROS homeostasis.

It has been reported that certain antioxidants such as quercetin, honokiol, MitoQ, etc., are associated with mitochondrial biogenesis, membrane potential, and redox status, and can promote mitochondrial homeostasis by scavenging mtROS.^[^ ^113^, ^114^, ^115^ ^]^ However, their clinical application is hindered by low bioavailability and high metabolic rates. Therefore, utilizing carriers to construct smart composite materials for delivering these drugs can overcome these limitations effectively. For instance, employing CeO_2_ as a nanocarrier for quercetin enhances not only quercetin's ability to scavenge ROS but also exploits CeO_2_’s antioxidant catalytic activity. This enables the modulation of upstream signaling pathways activated by ROS by activating M2 macrophage polarization, ultimately maintaining mitochondrial homeostasis(Figure 6B).^[^ ^114^ ^]^ Recently, a ROS‐sensitive “self‐gated” nanodrug delivery system, MitoQ@PssL NPs, constructed using amphiphilic polymer nanoparticles (PssL NPs), has garnered attention.^[^ ^115^ ^]^ The mitochondria‐targeted antioxidant MitoQ possesses the capability to clear mtROS in situ and inhibit lipid peroxidation. Under high ROS conditions, PssL degrades and releases MitoQ, which downregulates ROS levels by inducing mitochondrial autophagy. Meanwhile, as ROS levels gradually decrease, PssL ceases degradation, leading to the cessation of MitoQ release, thereby maintaining redox homeostasis.

#### 4.4.4 Ca^2+^ Ion Channel Modulators

Accumulation and efflux of mitochondrial calcium ions (mitoCa^2+^) serve as crucial feedback regulatory factors for many functions such as mitochondrial metabolism and cell death.^[^ ^116^ ^]^ Under inflammatory conditions, mitoCa^2+^ overload can trigger the sustained opening of the mitochondrial permeability transition pore (mPTP), further exacerbating Ca^2+^ overload and increasing ROS, forming a vicious cycle.^[^ ^117^ ^]^ Therefore, maintaining intracellular Ca^2+^ homeostasis is crucial for reducing mtROS production.

Researchers have attempted to reverse mitochondrial dysfunction by targeting mitochondrial calcium channels using siRNA or peptides, but clinical side effects persist.^[^ ^118^ ^]^ Additionally, chronic inflammation not only leads to mitoCa^2+^ overload but also activates the Wnt/β‐catenin pathway, inhibiting mitochondrial autophagy and disrupting its ROS‐clearing function. Damaged mitochondria accumulate continuously in cells, hindering cell differentiation.^[^ ^119^ ^]^ In light of this, Jin et al. developed a nano repair agent, METP NPs, responsive to intracellular microenvironments (esterases and low pH). They utilized positively charged mesoporous silica nanoparticles (TMAMSN) as the nanocarrier, and ethylene glycol‐bis(β‐aminoethyl ether)‐N,N,N′,N′‐tetraacetic acid (EGTA, a calcium chelator)/triphenylphosphine (TPP, a mitochondrial targeting agent) as the composite shell to form METP NPs. It enhances the targeting and flexibility of drug action by capturing excess Ca^2+^ to prevent mitoCa^2+^ overload. Simultaneously, under cellular microenvironment response, METP NPs release siRNA to inhibit Wnt/β‐catenin pathway dysregulation of mitochondrial autophagy, restoring cellular mitochondrial health(Figure 6C).^[^ ^119^ ^]^ This dual‐functional METP NPs hold promise as an effective therapeutic approach for chronic inflammation‐related diseases.

Recently, an inflammatory targeting nanosystem using hollow mesoporous dopamine nanoparticles (HMPDA) as a carrier has been described, namely HMPDA@BA/NAD+@LSA, which has multiple mechanisms to regulate cellular behavior(Figure 6D).^[^ ^120^ ^]^ Under the guidance of inflammation‐targeting peptide PEG‐LSA and inflammatory acidic microenvironment, it undergoes disassembly and simultaneously releases HMPDA, Ca^2+^ chelator BA‐AM, and NAD^+^. By clearing ROS, chelating excess mitoCa^2+^, and supplementing NAD^+^ in the mitochondrial energy pool, it precisely interrupts the transmission of three major pathways of cell necrosis, inflammation, and mitochondrial apoptosis from the source, thus rescuing mitochondrial function effectively. The synthesis of this drug provides a simple and effective co‐administration nano system, achieving efficient anti‐cell necrosis and anti‐inflammatory effects, with significant potential in antioxidant therapy.

## 5 ROS‐Generating Nanoplatforms

Due to their high oxidation potential, ROS can oxidize many cellular components, such as proteins and DNA, leading to cellular dysfunction and programmed or unprogrammed cell death. Importantly, the accumulation of ROS leads to alterations in antioxidant capacity and redox dynamics. From radiotherapy (RT) to photodynamic therapy (PDT) and many other ROS generation mechanisms, the use of ROS as a direct or indirect bactericidal or cancer cell‐destructive therapeutic strategy has been widely explored. In the following sections, we will delve into the mechanisms of ROS generation and explore nanoplatforms capable of modulating intracellular ROS production(Figure 2).

### 5.1 Endogenous Stimulus‐Responsive Nanoplatforms

In recent years, endogenous stimulus‐responsive nanoplatforms have gradually been applied in the biomedical field. These platforms release drugs in response to physiological conditions within cells, enhancing therapeutic effects while reducing side effects.^[^ ^20^ ^]^ For example, acid‐activatable nanoplatforms, a class of NPs triggered by H^+^ to catalyze the production of ROS. As mentioned earlier, Fe NPs exhibit pH‐responsive enzymatic activity. Scholars have designed pH‐activated biomimetic catalysts, such as Dex‐NZM, by encapsulating Fe_3_O_4_ NPs with dextran.^[^ ^121^ ^]^ Under the acidic pH of microbial infection, Dex‐NZM exhibits strong POD mimic activity, rapidly catalyzing the generation of OH• from H_2_O_2_ in situ. Additionally, the dextran coating promotes the binding of NZM to bacterial biofilms, enhancing its bactericidal effect.

**Figure 7:**
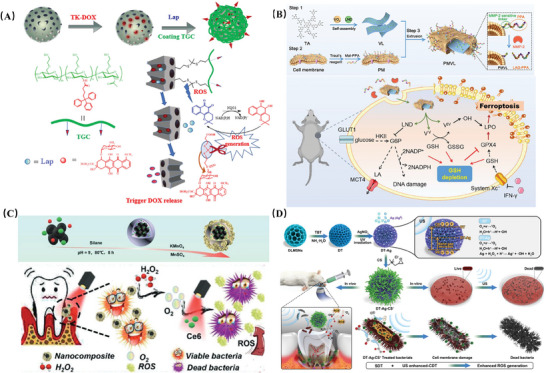
A) The preparation and work process of nanocarriers. Adapted with permission.^[^ ^123^ ^]^ Copyright 2022, Elsevier. B) Schematic Illustration Displaying the Preparation Process, Nanostructure, and MMP‐2 Responsive Behavior of PMVL. Adapted with permission.^[^ ^125^ ^]^ Copyright 2023, American Chemical Society. C) Schematic illustration of the synthesis of F@Ce6‐M NCs and alleviating periodontal pocket hypoxia and enhancing aPDT by F@Ce6‐M NCs decomposition of H_2_O_2_ to produce O_2_•−. Adapted with permission.^[^ ^126^ ^]^ Copyright 2021, John Wiley and Sons. D) Schematic illustration for the preparation and the anti‐periodontitis mechanism of DT‐Ag‐CS^+^ nanoparticles. Adapted with permission.^[^ ^127^ ^]^ Copyright 2022, Elsevier.

Literature suggests that changes in ROS levels in oxidative damage environments may be more indicative than pH fluctuations. Therefore, utilizing ROS‐responsive polymers to disrupt cellular redox homeostasis holds promise as an antibacterial and anticancer strategy. ROS‐responsive NPs typically contain elements and groups such as sulfur, boron, and tellurium that are easily oxidized or cleaved by ROS.^[^ ^104^, ^122^ ^]^ For example, the nanoplatform M‐TDOX/Lap@TGC possesses self‐regulating ROS levels. This NP conjugates doxorubicin (DOX) to MSNs via a thioether bond (TK). With increasing environmental ROS, the TK bond is activated, releasing the ROS inducer β‐Lapachone (Lap), which subsequently enters cancer cells and specifically responds to endogenous ROS, inducing mitochondrial dysfunction. This promotes the amplification and accumulation of surrounding ROS circulation, triggering tumor‐specific DOX release and inducing cancer cell apoptosis. This dual ROS‐responsive NP achieves efficient anticancer effects(**Figure** **7**A).^[^ ^123^ ^]^

Similarly, utilizing aberrantly expressed enzymes as “gates” for drug release provides a new approach for designing enzyme‐responsive nanoplatforms. Compared to other endogenous stimuli, enzyme‐triggered responses offer advantages such as mild conditions, high catalytic efficiency, and strong substrate selectivity.^[^ ^104^ ^]^ Taking hyaluronidase (HAase) as an example, it is often overexpressed in infection and cancer microenvironments, continuously degrading a large amount of hyaluronic acid (HA) in the extracellular matrix (ECM), leading to irreversible ECM disruption and ultimately promoting disease progression. Inspired by this, researchers have constructed HAase‐responsive antibacterial or anticancer drug delivery nanoplatforms based on the specific degradation of HA. For example, the composite nanoparticle PCN‐224‐Ag‐HA, coated with HA, undergoes degradation under the action of HAase secreted by bacteria at the lesion site, triggering the release of Ag^+^ and achieving enzyme‐responsive controllable sterilization.^[^ ^124^ ^]^

However, the intersection of various internal and external factors makes the pathological microenvironment extremely complex. Single‐responsive nanoplatforms are insufficient to cope with this complexity. To provide precise and rapid stepwise cascade reactions to enhance chemodynamic therapy (CDT), the design and preparation of dual‐responsive and multi‐responsive nanomaterials become necessary. For example, the enzyme and pH dual‐responsive nanosystem PMVL, which is camouflaged with tumor cell membranes modified with programmed death ligand 1 (PD‐L1) inhibitory peptide (PPA) and coupled with matrix metalloproteinase‐2 (MMP‐2) sensitive peptide(Figure 7B).^[^ ^125^ ^]^ In response to the overexpression of matrix metalloproteinase‐2 (MMP‐2) in cancer cells, first, PPA is released to block the PD‐1/PD‐L1 recognition between cytotoxic T lymphocytes (CTLs) and tumor cells. Second, the remaining part of PMVL decomposes under tumor acidic conditions, releasing V^4+^, V^5+^, and LND. On one hand, V^4+^ mediates Fenton‐like reactions to produce a large amount of ROS, and on the other hand, the anticancer drug LND inhibits both glycolysis (reducing ATP supply) and the pentose phosphate pathway (reducing NADPH production), as well as the valence state transition of V^5+^ and V^4+^, and inhibition of the Xc‐ system, leading to the consumption of reducing GSH and deactivation of GPX4. Meanwhile, the inhibition of glycolysis alleviates the acidic tumor microenvironment by reducing intracellular lactate production, preventing the polarization of immunosuppressive M2 macrophages.^[^ ^125^ ^]^ This multi‐responsive nanoplatform not only ingeniously constructs a cascade reaction system between various links, achieving self‐sufficiency of intermediate products, but also overcomes the diffusion barriers of drugs at the target site, improves the atomic economy of the overall reaction, and greatly enhances reaction efficiency. This provides a new direction for the application design of nanomaterials, holding tremendous potential in combating infections, injuries, cancers, and other conditions.

### 5.2 Exogenous Stimulus‐Responsive Nanoplatform

While endogenous stimulus‐responsive nanoplatforms can achieve targeted drug release and effects in various ways, human intervention often fails to effectively modulate in situ chemical reactions. Therefore, intervention with specific external energy fields during the CDT process can achieve temporal and spatial regulation of the reaction to a certain extent, accelerating Fenton‐like reactions and further promoting ROS generation to enhance therapeutic effects.^[^ ^33^ ^]^

PDT is a widely used technique for designing nanoplatforms for treating infections and cancer. Under irradiation at specific wavelengths, photosensitizers absorb light energy, undergo energy level transitions, and generate electrons that combine with surrounding oxygen to produce various ROS molecules, oxidizing biomacromolecules within cells and inducing death in bacteria or tumor cells. Among them, photosensitizers, as the core materials of PDT, determine its conditions of use and treatment efficiency. The most commonly used photosensitizers include porphyrins, phthalocyanines, and their derivatives. For example, intelligent nano‐composite materials F@Ce6‐M NCs, designed using the photosensitizer molecule Chlorin e6 (Ce6) with self‐generating oxygen functionality, have been developed.^[^ ^126^ ^]^ Under near‐infrared light (NIR) irradiation at the infection site, Ce6‐mediated PDT induces microbial death by generating ROS. Notably, surface‐modified MnO_2_ provides catalase‐like activity, sustainably supplying O_2_ for aPDT, significantly enhancing the biological effects(Figure 7C).

Similarly, ultrasound sensitizer‐assisted sonodynamic therapy (SDT), due to its non‐invasive, locally targeted, and high tissue penetration capabilities, holds promise for a wide range of applications in deep bacterial infections and cancer treatment. For instance, a novel nanosonosensitizer DT‐Ag‐CS^+^ has been developed, consisting of mesoporous TiO_2_ nanoparticles (DT), Ag, and quaternary ammonium chitosan (CS^+^)(Figure 7D). Under US stimulation and reaction with H_2_O_2_, the positively charged CS^+^ released by DT‐Ag‐CS^+^ enhances penetration into bacterial cells, while the e^−^/h^+^ separation ability of Ag+ significantly improves SDT performance, catalyzing the production of more OH• and ^1^O_2_, demonstrating significant antibacterial effects.^[^ ^127^ ^]^

The literature indicates that when the local tissue temperature reaches a certain range, it can accelerate the rate of the Fenton reaction and ionization processes, achieving the synergistic effect of photothermal therapy (PTT) and CDT.^[^ ^128^ ^]^ As a typical example, the multifunctional nano‐platform BSArGO@ZIF‐8 NSs, combined with ion interference and PTT, has demonstrated anticancer effects. On one hand, this nano‐platform induces intracellular Zn^2+^ overload to elevate ROS levels, initiating mitochondrial damage and cell apoptosis mediated by the pro‐apoptotic protein bim. On the other hand, the photothermal effect of reduced graphene oxide (rGO) not only increases the local temperature to enhance the Fenton reaction but also synergistically activates cell apoptosis events mediated by Zn^2+^ interference, thereby promoting CDT‐induced cancer cell apoptosis.^[^ ^129^ ^]^ Subsequently, nano‐platforms combining PTT and SDT emerged, such as CRDAs. Under dual irradiation of NIR and US, CRDAs release photon‐active ultra‐small Cu_2_xS NPs and the sonosensitizer Rose Bengal (RB), converting incident light energy into heat to promote charge transfer and mediate the generation of numerous free radicals, achieving in situ PTT and SDT effects for dual attack on tumors.^[^ ^130^ ^]^ This provides new insights and perspectives for the future development of multifunctional CDT agents.

### 5.3 Micro‐Nanorobots, MNRs

Nanorobots, as emerging materials in the field of nanomedicine, have garnered increasing attention. Artificial micro‐nanorobots, also known as micromotors, are intelligent propulsion devices operating at the micro and nanoscale, capable of converting external environmental energy (such as light, magnetic, and electrical energy) into mechanical energy.^[^ ^131^, ^132^, ^133^, ^134^ ^]^ The objective of self‐propelled nanorobots is to overcome the limitations of traditional nanomedicine. For instance, micro‐nanorobots can provide real‐time disease detection and monitoring,^[^ ^135^, ^136^, ^137^ ^]^ targeted therapy,^[^ ^138^, ^139^ ^]^ or on‐demand release of drugs, active substances, and antimicrobial agents.^[^ ^140^, ^141^ ^]^ Leveraging their small size and flexible movement, they hold promising prospects in various fields such as micro‐nano manipulation and minimally invasive surgery.^[^ ^142^ ^]^

**Figure 8:**
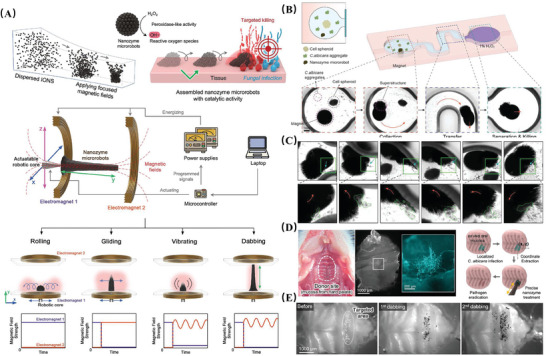
A) Assembly, control, and functional properties of robotic nanozyme assemblies and their mode of action. B) A schematic of the experimental platform for testing C. albicans capture and killing in the presence of cell spheroid using nanozyme microrobots. C) Fungal binding, dragging, and engulfing by the nanozyme microrobot. Close‐up images show the fungal aggregates marked by green lines. D) Focal C. albicans infection developed on the oral mucosa characterized by localized hyphal accumulation, and schematic diagram of the coordinate extraction and precision‐guided treatment using the dabbing nanozyme microrobot. E) Bright field images showing sequential nanozyme dabbing. Adapted with permission. Copyright 2023, John Wiley and Sons.^[^ ^140^ ^]^

Among them, magnetically driven micro‐nanorobots exhibit excellent biocompatibility, remote operability, recyclability of magnetic materials, and ease of manipulation, making them widely applicable in the biomedical field.^[^ ^143^, ^144^, ^145^, ^146^ ^]^ The incorporation of magnetic materials (such as Fe_3_O_4_, Fe, Ni, etc.) provides the motion system with specific magnetic operability, while also regulating ROS and initiating the degradation process of external energy fields to improve treatment efficacy. For example, the Catalytic Antimicrobial Robots (CARs) developed by Koo's team are capable of performing multiple tasks targeting both pharmacological and mechanical resistance factors, effectively eliminating dental plaque biofilms.^[^ ^147^ ^]^ Under gradient magnetic field control, CARs employ both helical and blade shapes to penetrate and remove biofilms from cylindrical tube walls, generating ROS with IONP‐like peroxidase activity, degrading EPS, subsequently killing bacteria, and physically removing degradation products. Compared to traditional biofilm removal methods, CARs leave no residual traces of biofilm, significantly reducing the possibility of biofilm regrowth. Subsequently, the team developed a nanozyme microrobot platform with enhanced precision based on this foundation, capable of accurately directing catalytic action to the site of infection for rapid bactericidal effects. The platform utilizes electromagnetic field frequency modulation and spatiotemporal control to form iron oxide nanozyme components with dynamic shape transformation and catalytic activation functions. Compared to freely dispersed IONPs, the nanozyme microrobots exhibit reduced catalytic activity in a static state due to limited surface area, thereby suppressing unnecessary catalytic reactions. On the other hand, the nanozyme microrobots can perform localized catalytic reactions when in motion, providing an effective means of generating and directly delivering ROS to the target spatial location through dynamic movement, thus avoiding off‐target effects(**Figure** **8**).^[^ ^140^ ^]^ This approach offers an effective targeted therapeutic method for the elimination of pathogens at the site of infection. Additionally, light‐driven nanorobots also demonstrate advantages, with such MNRs exhibiting different motion behaviors depending on the wavelength of light waves. Moreover, the modification of NPs can enhance their ability to generate and release ROS, thereby initiating the photodegradation process and displaying superior oxidative stress amplification effects.^[^ ^15^ ^]^

Recently, FeO@mSiO_2_/Au‐CAT Janus self‐propelled nanocatalytic robots (JNCRs) with PTT effects have attracted considerable attention.^[^ ^148^ ^]^ JNCRs, exhibiting CAT‐like activity, provide propulsion for their autonomous movement, catalyzing the generation of O_2_ from H_2_O_2_ to improve the tumor's hypoxic environment, thereby enhancing tissue penetration. Particularly, the modification of Fe and Au endows JNCRs not only with photothermal conversion effects under light stimulation but also with pH‐responsive iron release and ROS generation capabilities, significantly enhancing the anticancer efficacy of PTT and CDT. Despite this, MNRs are still in their infancy, with a long way to go before clinical translation. However, with continued research and development, the enormous potential of MNRs will bring positive and far‐reaching impacts to human society in terms of life, healthcare, and well‐being at the micro and nanoscale.

## 6 Applications of ROS‐Based Nanomaterials in Dentistry

**Figure 9:**
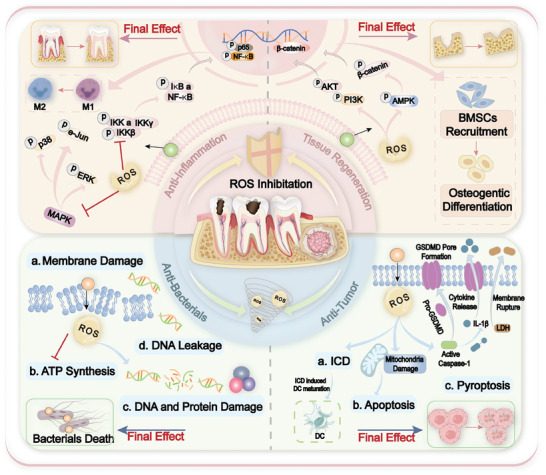
Oral diseases caused by dysregulation of ROS generation and scavenging and treatment strategies. Inflammatory conditions and tissue impairments are significantly impacted by ROS overproduction, which can be mitigated through antioxidant therapy induced by nanomaterials. Conversely, infectious diseases and tumors can exploit ROS‐generating nano materials to induce cytotoxic effects, offering potential therapeutic avenues.

Oral diseases such as dental caries, periodontal disease, and oral cancer can cause significant harm to human health. Although their specific pathogenic mechanisms vary, oxidative stress and inflammation have been identified as key mediators of these diseases.^[^ ^40^, ^148^ ^]^ On the one hand, amplifying oxidative stress can help the host combat microbes and cancer cells, demonstrating excellent therapeutic potential. On the other hand, targeting excessive ROS to block aberrant inflammatory responses is considered a feasible anti‐inflammatory strategy, with numerous nanomedicines carefully designed to possess ROS scavenging capabilities and achieve desirable anti‐inflammatory effects. Given the multifaceted regulatory roles of the aforementioned nanomaterials in cellular behavior, ROS‐based nanomedicines are regarded as an attractive option for delivering oral therapeutic agents. Moreover, some nanozymes with natural enzyme‐mimicking activity can directly scavenge or generate excessive ROS. Here, we illustrate the application of nanomaterials regulating oxidative‐reductive signaling in dentistry, aiming to facilitate the further development of ROS‐based nanotherapy in both basic research and clinical applications (**Figure** **9**).

### 6.1 Oral Biofilm Infection

The human oral cavity is a microbial swamp prone to dysbiosis under the repeated stimulation of various internal and external factors, leading to a range of oral diseases.^[^ ^149^ ^]^ Among these, the formation of biofilms is a major virulence factor underlying chronic oral infections such as caries, apical periodontitis, and implant‐related infections. While conventional antimicrobial agents or dental fillings can partially control the development of biofilm infections, the multifactorial nature of biofilm development and drug tolerance pose significant challenges to the use of traditional antibiotics.^[^ ^150^, ^151^, ^152^ ^]^ Hence, novel approaches to prevent and treat infections while enhancing multifunctionality are imperative. It has been reported that during microbial infections, the host immune system can produce relatively nonspecific effector molecules to suppress pathogen replication, such as ROS.^[^ ^153^ ^]^ Inspired by this, researchers have developed ROS‐generating nanoplatforms that, by interfering with electron transfer or producing reactive metabolic byproducts, not only disrupt cellular energy metabolism but also upregulate intracellular ROS to disrupt bacterial redox metabolism, achieving bactericidal effects.^[^ ^17^, ^154^ ^]^

**Figure 10:**
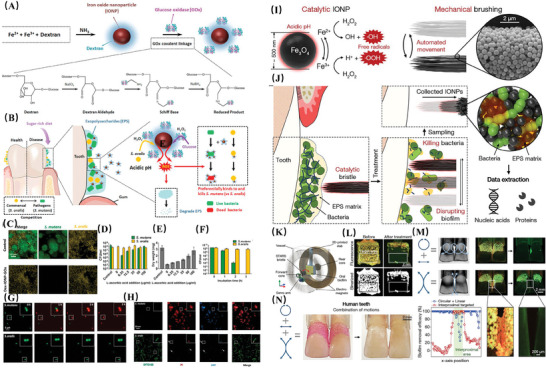
A) Schematic depiction of the synthesis of the Dex‐IONP‐ GOx nanohybrid. B) Schematic depiction of the selective catalytic–therapeutic mechanism of Dex‐IONP‐ GOx for treatment of virulent acidogenic biofilms. C) FISH microscopy of vehicle control treated biofilm (top row) and biofilm treated with Dex‐IONP‐GOx (bottom row). D,E) Effect of IONP‐GOx on bacterial viability(D) and the mass of biofilm(E) after addition of different concentrations of l‐ascorbic acid. F) Effect of Dex‐IONP‐ GOx on bacteria viability over time (*n* = 3). G) Micrographs of bacteria incubated with Dex‐IONP‐ GOx at different time points. H) Detection of ROS in *S mutans* or *S. oralis* after treatment with Dex‐IONP‐ Gox. Reproduced with permission from Ref.[42]. I) IONPs are multifunctional with peroxidase‐like activity, generating free radicals at the site of mechanical cleaning providing both antimicrobial treatment and physical biofilm removal. J) Bristle motion is controlled to disrupt biofilms through mechanochemical action and retrieve biofilm contents for diagnostic sampling. K) A schematic of the experimental platform for measuring efficacy of biofilm cleaning. L) Biofilm cleaning efficacy is evaluated across the targeted area. M) Fundamental motion patterns are tested on human tooth mimics. Before and after comparisons of fluorescently labeled biofilms cleaned with a combination of circular and linear motions (top) and targeted interproximal cleaning (middle) demonstrating efficacy on complex topographies. Circular motions effectively remove the bulk of biofilm from the facial tooth surfaces, while targeted motion selectively removes biofilm from interproximal space (bottom, left). N) Combined motions demonstrate complete biofilm removal on ex vivo human tooth. Adapted with permission.^[^ ^138^ ^]^. Copyright 2022, American Chemical Society.

As a typical example, the Koo team and others combined Fe_3_O_4_ NPs with H_2_O_2_ to develop a nanozyme, CAT‐NP, for the first time used in the treatment of dental caries.^[^ ^12^ ^]^ In the acidic ecological niche of dental biofilm, the inherent POD‐like activity of CAT‐NP facilitates the catalysis of the generation of highly oxidative OH•, thereby triggering the degradation of protective bacteria ECM and the death of Streptococcus mutans, effectively inhibiting the development of dental caries. However, the antibacterial effect of CAT‐NP is limited due to the difficulty in ensuring the content of H_2_O_2_ and the local acidic pH environment, which may lead to a reduction in sterilization efficacy. To address this issue, the Koo team later covalently coupled glucose oxidase (GOx) with dextran‐coated IONP (Dex‐IONP) and designed a dual‐functional nano‐hybrid system, Dex‐IONP‐GOx, for the removal of dental biofilms (**Figure** **10**A,B). In the presence of glucose, GOx can decompose glucose into H_2_O_2_ and gluconic acid, not only addressing the deficiency of endogenous H_2_O_2_ but also providing a homogeneous acidic environment. Through IONP, H_2_O_2_ is decomposed into OH•, achieving efficient sterilization (Figure 10C–H).^[^ ^42^ ^]^ In another report, Yang et al. introduced copper‐doped carbon dots (Cu‐CDs) as a nano mouthwash for eliminating oral pathogenic bacteria.^[^ ^155^ ^]^ Cu‐CDs possess oral‐adaptive dual‐enzyme mimic catalytic activity (CAT and POD), continuously producing O_2_ and ROS through mimicking the cascaded catalytic reactions in the body, thereby inhibiting the adhesion of Streptococcus mutans and eradicating biofilms, achieving antibacterial, wound infection prevention, and teeth whitening purposes. Recently, H_2_O_2_ self‐supplying nano hydrogel Fe_3_O_4_‐CaO_2_ has been used for the removal of root canal biofilms.^[^ ^156^ ^]^ Triggered by H^+^, the encapsulation of the hydrogel enables CaO_2_ to slowly decompose to generate H_2_O_2_, which is then catalyzed by IONP with POD‐like activity to produce OH•, inducing a strong anti‐biofilm effect. However, in this system, the authors overlooked the ability of CaO_2_ release to disrupt cellular calcium homeostasis and induce mCa^2+^ overload damage, which would induce cell apoptosis to promote sterilization. This should also be an ideal breakthrough in combating infections.

Furthermore, the application of nanomaterials controlled by external energy fields in dentistry has been widely reported. For example, Li's team designed a series of polymer implants with piezoelectric surfaces (piezoPolymer) for the treatment of implant‐associated infections (IAI) by constructing metal/piezoelectric heterojunction structures on the surface of dental implants.^[^ ^157^ ^]^ Under ultrasonic stimulation, the piezoelectric nano membranes (piezoPCL film) embedded in the implants can promote piezoelectric charge transfer at the interface between bacteria and implants to produce local ROS, thereby inhibiting the survival of Staphylococcus aureus by interfering with biofilm stability, transmembrane transport, and carbon metabolism, providing an on‐demand non‐invasive treatment method for IAI. Meanwhile, a root canal filling material with piezoelectric surface, namely piezoGP, was developed, confirming that piezoGP can induce piezoelectric catalytic antibacterial action through ultrasonic irradiation, effectively preventing root canal reinfection caused by leakage of the sealed space between the gum and root canal. Moreover, even in the event of root canal reinfection, only ultrasound irradiation is needed for clearance. In addition, the Koo team utilized IONP to construct a surface morphology‐adaptive robot upper structure (STARS) for the removal of oral biofilms (Figure 10I,J). STARS not only has the characteristics of shape adaptation and self‐assembly on the biofilm surface but also achieves antibacterial effects similar to “toothbrush” and “dental floss.” The research indicates that under externally applied magnetic field‐controlled positioning, IONP contained in STARS exhibits POD mimetic activity, effectively catalyzing H_2_O_2_ to produce ROS to degrade biofilms, achieving precise and efficient antibacterial effects (Figure 10K–N).^[^ ^138^ ^]^

### 6.2 Oral Inflammatory Diseases

Oral chronic inflammatory conditions (such as periodontitis, peri‐implantitis, and oral ulcers) are mostly caused by infections and immune dysregulation. In the early stages of inflammation, a large number of polymorphonuclear neutrophils (PMNs) are recruited and activated in the pathological area. They release ROS to eliminate pathogens, with excess ROS cleared by the body's antioxidant protection system to prevent further tissue damage. However, in most cases, due to prolonged chronic stimulation during inflammation, immune cells produce ROS while killing pathogenic microorganisms, disrupting the redox balance. On the other hand, excessive ROS leads to mitochondrial dysfunction and a series of abnormal enzyme‐catalyzed reactions, further stimulating ROS production, thus forming a sustained oxidative stress cycle that exacerbates inflammatory damage.^[^ ^158^, ^159^ ^]^ Therefore, NPs with ROS scavenging ability and regulation of oxidative stress‐related factors are potential candidates for restoring redox homeostasis and effectively treating oral inflammatory diseases.^[^ ^160^ ^]^

**Figure 11:**
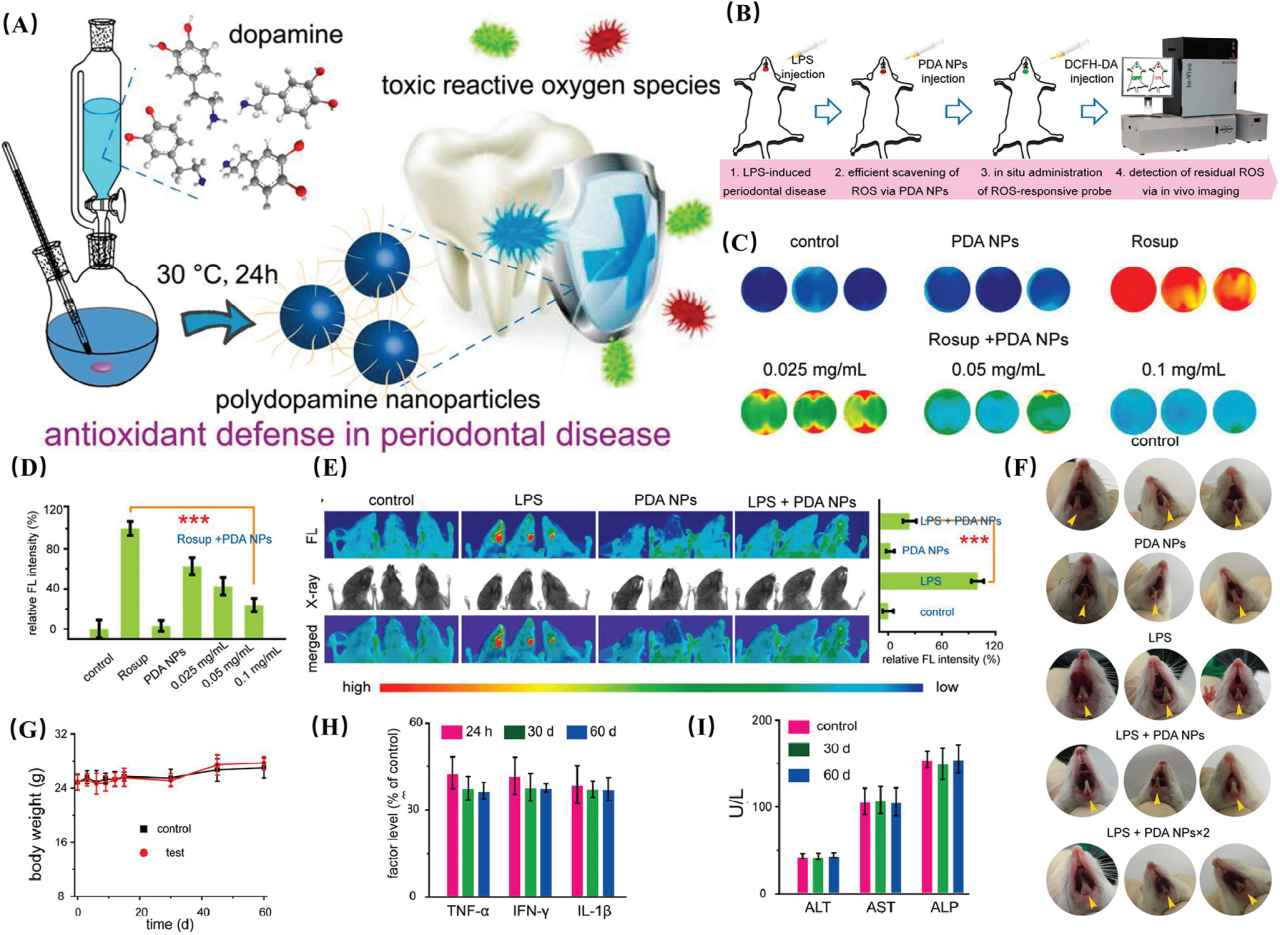
A) Schematic illustration of the typical synthesis of PDA NPs and their usages as efficient ROS scavengers in periodontal disease. B) Schematic illustration of LPS‐induced periodontal disease in BALB/c nude mice and relative experimental design of ROS scavenging. C,D) In vitro and E) in vivo fluorescence imaging of ROS removal capacity and relative quantitative information via PDA NPs by using an in vivo imaging system as the test facility. F) Photographic images of Kunming mice 3 days after various treatments. Changes in G) mouse body weights; H) cytokine response in mice; and I) serum levels of ALT, AST, and ALP in mice. Adapted with permission.^[^ ^77^ ^]^ Copyright 2018, American Chemical Society.

As a typical example, The Yang team utilized PDA NPs as intelligent scavengers to design and construct an efficient antioxidant defense platform for the clearance of ROS induced by oxidative stress in periodontal disease (**Figure** **11**A). This study provides detailed insights into the antioxidative role and underlying mechanisms of PDA NPs in periodontal disease, while offering new perspectives for the development of safe and effective antioxidant defense platforms (Figure 11B–I).^[^ ^77^ ^]^ Recently, Kim et al. first reported a diatomaceous earth‐doped MnO_2_ nanozyme, used for the treatment of peri‐implantitis.^[^ ^161^ ^]^ Mn^4+^ exhibits catalase‐like activity, catalyzing the decomposition of H_2_O_2_ into H_2_O and O_2_, effectively alleviating peri‐implant inflammation. Additionally, the Gao team developed VB_2_‐modified IONzymes for the treatment of oral ulcers. VB_2_‐modified IONzymes significantly enhance antioxidant enzyme activity, promoting ROS clearance, thereby effectively protecting mucosal cells from H_2_O_2_ damage and accelerating oral ulcer healing.^[^ ^162^ ^]^

**Figure 12:**
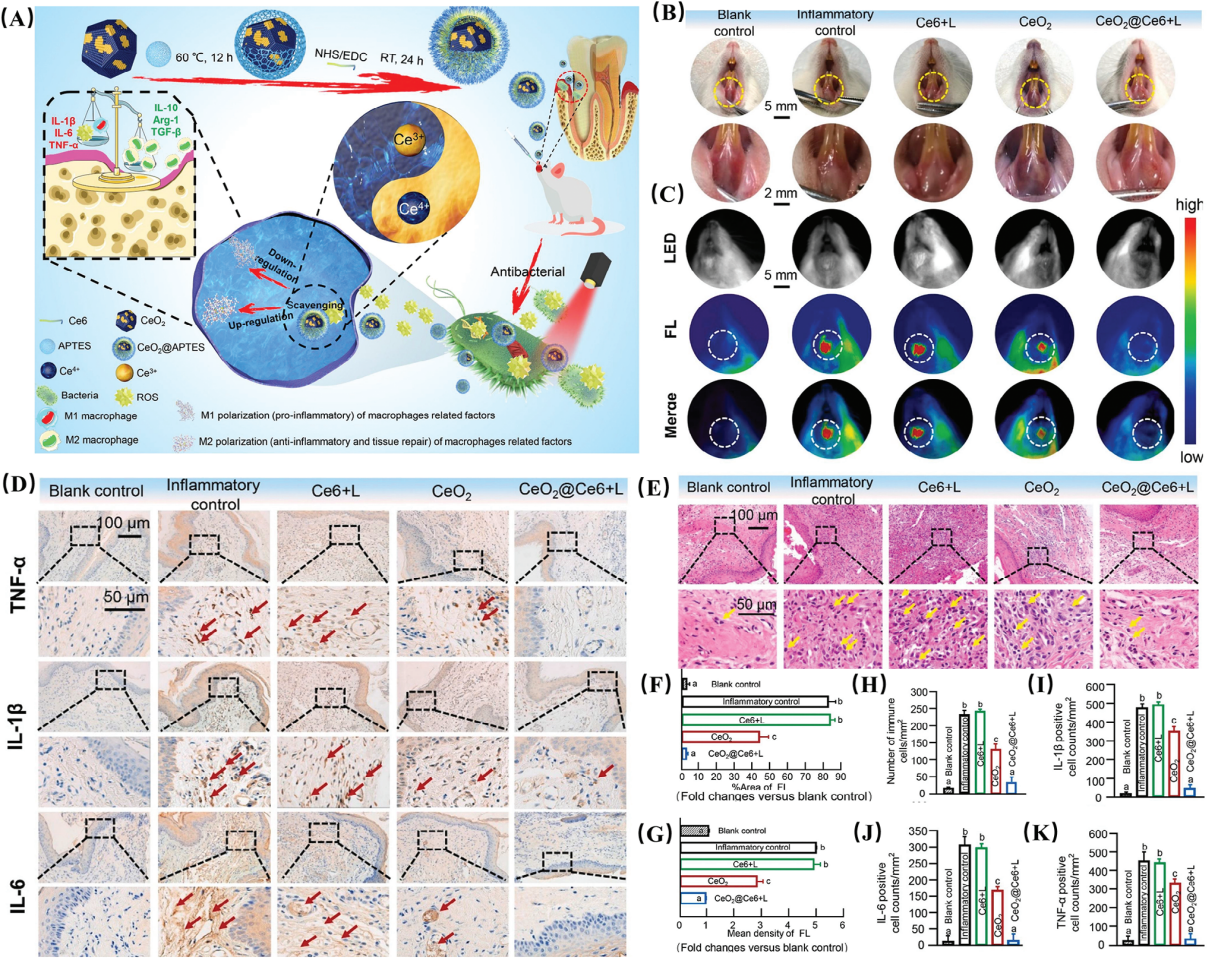
A) Schematic illustration of CeO_2_@Ce6 nanocomposite in synthesis, the antibacterial mechanism and modulating the polarization of macrophages for the treatment of periodontal diseases. B) The intraoral photos for modeling rats after 4‐day treatments by different nanoparticles. C) In vivo fluorescence imaging of ROS level at the site of periodontal infections with different treatments (from top to bottom: images captured at bright field, fluorescence activation and the combination of above). F,G) The corresponding quantification for local ROS level by mean density and area of fluorescence. D,E) Histological and Immunohistochemical evaluation of periodontal tissues after 4‐day treatments. H) The corresponding quantification of immune cells in H&E staining sections. I–K) The corresponding quantification of positive cells with (K) TNF‐α, (I) IL‐1β, and (J) IL‐6 immunostaining.  Adapted with permission.^[^ ^163^ ^]^ Copyright.2021, Elsevier.

Although nanozyme‐mediated regulation of redox homeostasis has made some progress in the treatment of oral inflammation, a single catalytic process is still insufficient to restore the complex oral inflammatory microenvironment. With the depletion of substrates, the catalytic reactions induced by nanozymes will be unsustainable. In this case, the development of cascade nanozymes with multi‐enzyme activity has shown potential to enhance catalytic therapeutic effects. For example, Dong et al. have developed a multifunctional nanocomposite material, ceria@Ce6, which achieves bactericidal and anti‐inflammatory effects through dual‐targeted modulation (**Figure** **12**A). This nanoplatform utilizes antimicrobial photodynamic therapy (aPDT) during the first stage, with red light irradiation, for antibacterial purposes. Subsequently, it employs the dual antioxidant enzyme‐like activity of nanoceria to eliminate residual ROS, thereby downregulating M1 polarization (pro‐inflammatory) and upregulating M2 polarization (anti‐inflammatory and regenerative) of macrophages to modulate host immunity. This work addresses the most challenging issue in clinical application of aPDT, namely the contradiction between ROS bactericidal action and inflammation. The structural design of this nanocomposite confers highly rational and effective ROS modulation capability post‐aPDT, achieving a perfect combination of bactericidal and anti‐inflammatory effects (Figure 12B–K).^[^ ^163^ ^]^

Similarly, the integrated cascade nanozyme Pt@PCN222‐Mn, which can mimic the cascade catalytic reaction system in vivo, has been used to reshape the inflammatory microenvironment of temporomandibular joint osteoarthritis (TMJ OA).^[^ ^164^ ^]^ Pt@PCN222‐Mn possesses both SOD and CAT mimic activities. Through SOD activity, it clears O_2_^•−^ to generate H_2_O_2_, which is then decomposed into H_2_O and O_2_ by CAT activity. Both in vitro and in vivo experiments have demonstrated that Pt@PCN222‐Mn can effectively inhibit the degradation of ECM and apoptosis of chondrocytes, ultimately delaying the progression of TMJ OA. Similarly, Han et al. reported on creating cobalt oxide‐supported Ir (CoO−Ir) as a cascade and ultrafast artificial antioxidase to alleviate local tissue inflammation and bone resorption in periodontitis.^[^ ^165^ ^]^ CoO‐Ir NPs provides not only efficient cellular protection from ROS attack but also promotes osteogenetic differentiation in vitro. This nanozyme‐based cascade catalytic therapy, where enzymes cleverly form a cascade reaction system, achieving self‐sufficiency of intermediate products and forming a closed‐loop reaction, greatly enhances reaction efficiency and represents a promising strategy for improving efficacy.

Furthermore, many nanomaterials, including nanozymes and non‐enzyme‐active NPs, are designed as carriers for other therapeutic agents to achieve synergistic therapeutic effects. For instance, a hollow mesoporous Prussian blue (HMPB) nanozyme functionalized with PDA was developed as a carrier for delivering curcumin (Cur) in a ROS‐responsive nano‐platform (HMPB@Cur@PDA) for the treatment of maxillofacial infections.^[^ ^166^ ^]^ The study demonstrates that under high ROS response, HMPB@Cur@PDA degrades to expose HMPB nanozyme, Cur, and PDA, which not only possess potent ROS scavenging ability but also enhance the antioxidant enzyme activity of Prussian blue through bonding interactions, synergistically halting the progression of inflammatory tissue damage, offering novel insights for the development of therapeutic strategies for maxillofacial infections. Similarly, utilizing a mesoporous Prussian blue metal‐organic framework (MPB) loaded with baicalin (BA), an antioxidant nano‐platform MPB‐BA was successfully developed for the treatment of periodontitis.^[^ ^167^ ^]^ Upon internalization into cells, MPB‐BA releases BA, which possesses antioxidant properties that promote the expression of antioxidant enzymes by upregulating Nrf2, thereby clearing excess ROS and alleviating periodontitis inflammation.

### 6.3 Oral Cancer

Oral squamous cell carcinoma (OSCC) is the most common malignant tumor of the head and neck region, and current primary treatment modalities still predominantly involve surgery, supplemented with standard radiotherapy or chemotherapy. OSCC presents with various risk factors, including excessive alcohol consumption, smoking, viral infections, and genetic predisposition, which may independently or synergistically influence tumor progression.^[^ ^168^ ^]^ It is noteworthy that the occurrence of all malignant tumors, including OSCC, is closely associated with the formation of the tumor microenvironment (TME), which determines tumor progression, metastasis, and drug resistance.^[^ ^169^ ^]^ However, the TME is intricate, with various microenvironmental features such as oxidative stress, low pH, hypoxia, and interactions between cellular microenvironments. Therefore, combination therapies targeting TME features are expected to achieve better treatment outcomes.^[^ ^170^, ^171^ ^]^

Research related to oral cancer indicates that, on one hand, cancer cells undergo metabolic reprogramming, upregulate antioxidant defense systems, increase mitochondrial oxidative phosphorylation (OXPHOS), and enhance nutrient uptake, leading to sustained proliferation.^[^ ^172^, ^173^, ^174^ ^]^ On the other hand, various byproducts of metabolic processes (such as lactate) and high levels of ROS produced from NADPH can cause irreversible damage and death of cancer cells.^[^ ^175^, ^176^ ^]^ Addressing these characteristics, ROS‐based nanotherapies show promise in the treatment of oral cancer by modulating ROS‐related metabolism within cancer cells using exogenous nanomaterials to disrupt their nutrient supply, trigger ROS production, and disrupt NADH homeostasis, selectively destroying cancer cells.^[^ ^177^, ^178^, ^179^ ^]^ For instance, a novel cholesterol‐modulating nanoplatform, PEG‐terbinafine‐Y8 nanoparticles (PYT NPs), developed by the Han team, synergistically stimulates immunotherapy and cholesterol metabolism regulation for OSCC treatment through PTT or PDT).^[^ ^180^ ^]^ Upon cellular internalization, PYT NPs release a cholesterol synthesis inhibitor (terbinafine) under laser irradiation, inducing Treg cell conversion and infiltration, leading to abnormal lipid metabolism in OSCC, effectively reversing immune suppression in the TME. Particularly, the photosensitizer Y8 released from PYT NPs exhibits excellent optical properties, inducing immunogenic cell death (ICD) of OSCC cells by generating high levels of ROS. The combined application of terbinafine and photodynamic therapy in this study explores a novel approach for treating OSCC with potential clinical translation.

**Figure 13:**
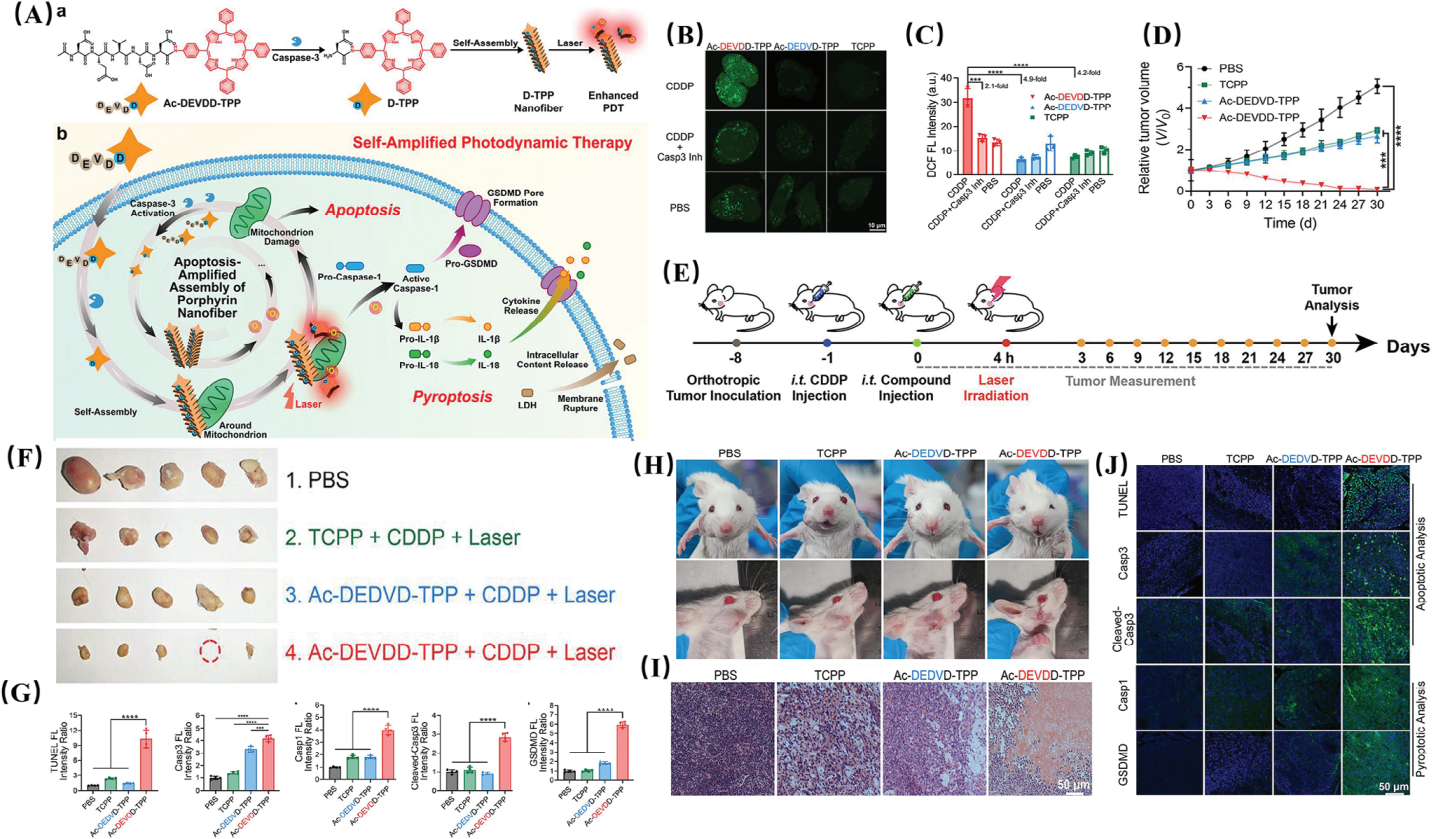
A) (a) Mechanism of Caspase‐3‐Activatable Ac‐DEVDD‐TPP for Enhanced Photodynamic Therapy (PDT); (b) Illustration of Self‐Amplified Photodynamic Therapy Enabled by Ac‐DEVDD‐TPP, Which Induced Enhanced Cell Apoptosis and Pyroptosis through Apoptosis‐Amplified Assembly of Porphyrin Nanofibers. B) CLSM images of SCC7 cells in f incubated with DCFH‐DA for 30 min after laser irradiation. C) Mean DCF fluorescence intensity of the cells in g. D) Growth curves of tumors on SCC7 orthotopic tumor‐bearing mice after various treatment. E) Schedule of orthotopic oral tumor implantation, CDDP preinjection, photosensitizer injection, laser irradiation, tumor measurement, and tumor analysis. F) Photographs of excised tumors at 30d. H) Photographs of the orthotopic tumors in mice. I) H&E staining of the tumor tissues at 30 d. G, J) Immunofluorescence staining of relative biomarkers of cell apoptosis and pyroptosis of tumors in SCC7 orthotopic tumor‐bearing mice after various treatments. Adapted with permission.^[^ ^183^ ^]^ Copyright 2023, American Chemical Society.

In addition, Wu et al. designed a hypoxia‐adaptive nanocomposite, TiO2@Ru@siRNA, for photodynamic immunotherapy in OSCC. These NPs can effectively facilitate the escape of HIF‐1α siRNA by causing lysosomal damage, thereby mitigating hypoxia. Notably, the TiO2@Ru@siRNA NPs raise cellular ROS levels through both oxygen‐dependent (Type II) and non‐oxygen‐dependent (Type I) pathways, thereby inducing pyroptosis and demonstrating a high level of PDT efficacy. Furthermore, PDT mediated by TiO2@Ru@siRNA effectively alleviates the immunosuppressive tumor microenvironment and enhances anti‐tumor immune responses.^[^ ^181^ ^]^ Similarly, the Liang team designed an intelligent peptide‐porphyrin molecule, Ac‐DEVDD‐TPP, based on the activation of the apoptotic protease cascade, achieving self‐amplifying photodynamic therapy for oral cancer (**Figure** **13**A). Studies have confirmed that upon entry into tumor cells, Ac‐DEVDD‐TPP, induced by light/drug, activates the apoptotic protease Caspase‐3, which subsequently cleaves the peptide substrate sequence DEVD to generate the triphenylphosphonium‐conjugated porphyrin molecule (D‐TPP). On one hand, D‐TPP can self‐assemble into nanofibers and aggregate around mitochondria. Under laser irradiation, these D‐TPP nanofibers generate more ^1^O^2^, inducing deeper cell apoptosis and activating more Caspase‐3, thus amplifying the apoptosis assembly process. On the other hand, while Ac‐DEVDD‐TPP promotes apoptosis of oral cancer cells, the excess generation of ^1^O^2^ activates the cell's Casp‐1‐dependent pyroptotic signaling pathway, achieving synergistic and efficient killing of oral cancer cells (Figure 13B–J).^[^ ^182^ ^]^ This cyclic amplification mechanism significantly enhances the apoptosis process of cancer cells, achieving an enhanced PDT effect on tumors.

However, the application of PDT‐mediated nanoplatforms is widely restricted due to high biotoxicity and poor targeting ability. In recent years, biobased nanotechnology has garnered attention for its ability to intelligently respond to pathological microenvironments by simulating the design and function of specific cells.^[^ ^183^ ^]^ For example, cell membrane camouflage technology enables nanoplatforms to acquire unique biological interface properties, reduce immune system absorption for prolonged circulation, and greatly enhance targeting. For instance, oral cancer cell membrane‐coated nanoparticles CM@Co‐Fc@HCQ.^[^ ^184^ ^]^ These NPs, loaded with hydroxychloroquine (HCQ) in a nano‐sized cobalt‐ferrocene metal‐organic framework (Co‐Fc), and coated with cancer cell membranes (CM). After internalization, Co‐Fc in CM@Co‐Fc@HCQ catalyzes the generation of a large number of ROS through the Fenton reaction, while HCQ inhibits the fusion of autophagosomes with lysosomes, reducing the clearance of ROS by tumor cells. The combined effects from various aspects result in the accumulation of ROS within tumor cells, ultimately inducing apoptosis. Particularly, the coverage of NPs by oral cancer cell membranes greatly enhances the tumor‐targeting effect of Co‐Fc@HCQ NPs, achieving an efficient killing effect on oral cancer cells.

### 6.4 Oral Soft and Hard Tissue Defects

Defects in oral and maxillofacial tissues caused by congenital (such as cleft lip and palate), age‐related, genetic factors, and acquired factors (such as tumor resection, trauma, inflammation) can lead to oral functional impairments, affecting the physical and mental health as well as the quality of life of patients.^[^ ^185^, ^186^ ^]^ Current treatment strategies mainly include mechanical debridement, antibiotics, and surgical tissue grafting.^[^ ^186^, ^187^, ^188^ ^]^ However, due to incomplete debridement, antibiotic resistance, high morbidity at donor sites, and a lack of tissue sources, the efficacy of these treatments remains unsatisfactory. Research indicates that during tissue healing, the defect area often experiences oxidative stress induced by elevated levels of ROS, leading to apoptosis and necrosis of stem cells and inhibiting their differentiation, thereby delaying tissue reconstruction.^[^ ^189^ ^]^ Therefore, modulating the ROS levels in the defect area to provide a favorable differentiation microenvironment for stem cells to promote tissue regeneration is a promising tissue repair approach.

**Figure 14:**
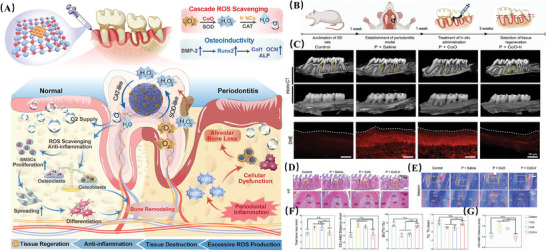
A) Mechanism of the CoO−Ir‐Based Artificial Antioxidases in Alleviating Inflammation and Bone Resorption to Combat Periodontitis. B) Experimental design for treating rat ligation induced periodontitis. C) Representative micro‐CT scanning bucco palatal images (upper panels), 3D reconstructed sections (lower panels) along the longitudinal direction of the maxillae;and Images of DHE fluorescence in periodontal tissue. The white line indicates the boundary of the periodontal tissue. D, G) H&E‐stained paraffin sections and the distance between CEJ and ABC. E) Masson's trichrome staining. F) Assessment of total bone loss of 9 loci, the distance between CEJ and ABC in mm, the percentage of BV/TV, and Tb. The between the upper first and second molars. Adapted with permission.^[^ ^166^ ^]^ Copyright 2023, American Chemical Society.

For example, novel ROS‐responsive NPs (PssL‐NAC) have been developed, targeting N‐acetylcysteine (NAC) as the drug of interest, which enhances the activity of endogenous GPx to downregulate intracellular ROS levels, alleviating the inflammatory microenvironment of hPDLSCs and promoting their osteogenic differentiation.^[^ ^41^ ^]^ In order to effectively modulate the high ROS and inflammation in the periodontal tissue microenvironment, Han et al. devised ultrafast artificial SOD mimetics and ultrafast CAT (CoO‐Ir) to alleviate local tissue inflammation and bone resorption in periodontitis (**Figure** **14**A). It was demonstrated that CoO‐Ir exhibits ultrafast SOD‐CAT cascade catalytic activity. Notably, CoO‐Ir showed significantly enhanced Vmax and TON values in H_2_O_2_ elimination, surpassing most artificial enzymes reported to date. Therefore, CoO‐Ir not only provides effective cellular protection against ROS attack but also promotes in vitro osteogenic differentiation (Figure 14B–G).^[^ ^165^ ^]^

**Figure 15:**
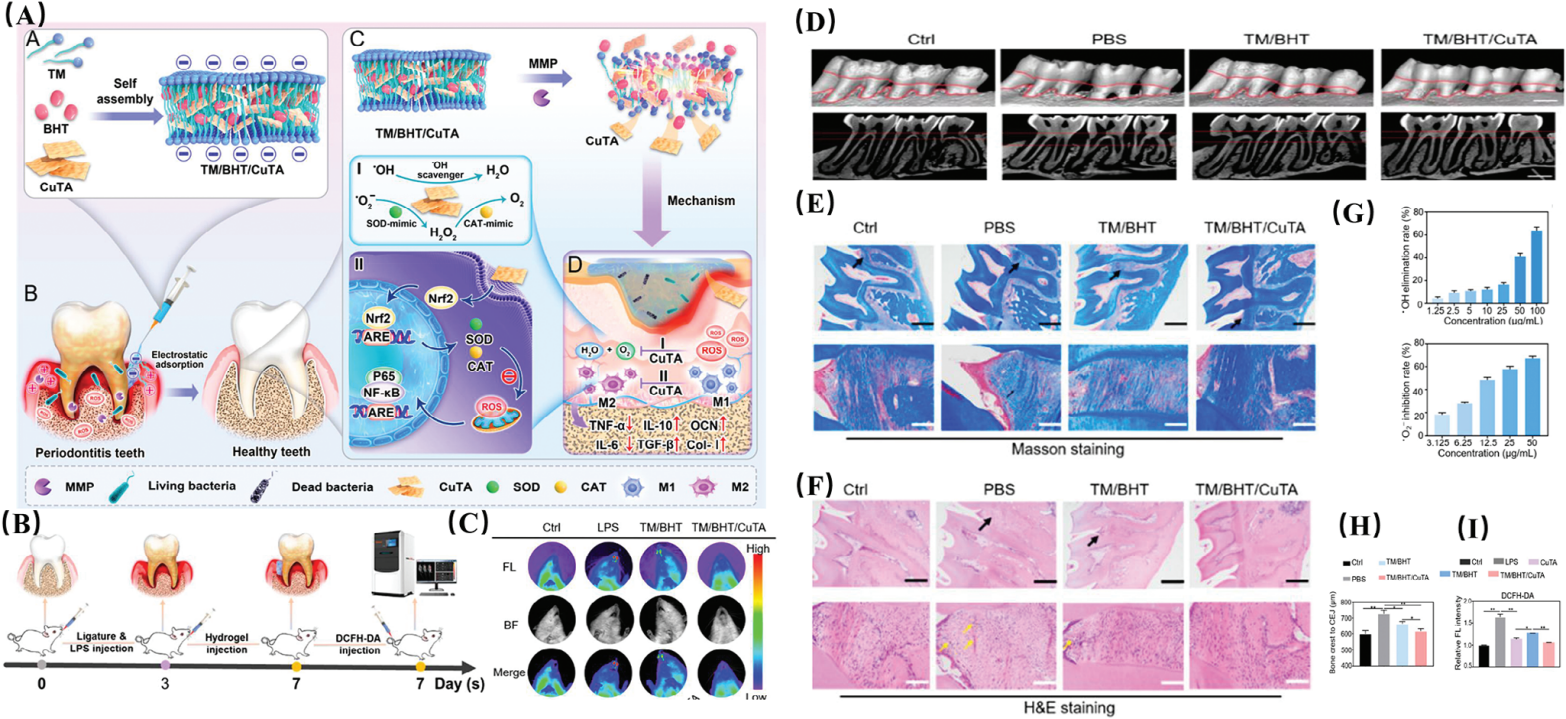
A) Schematic Illustration of the Synthesis of TM/BHT/CuTA Hydrogel and Its Application in Periodontitis. B) Sketch map of the establishment of the oxidative‐stress‐induced periodontitis by LPS. C) In vivo fluorescence images of ROS in infected periodontal tissue with different treatments. D) 3D reconstructed and the buccopalatal section images of the maxillary molars with different treatments by Micro‐CT. Redlines represent the distance between ABC and CEJ(scale bar = 1mm). E,F) Masson and H&E staining images of the periodontal tissue. G) OH• elimination and •O_2_− inhibition rates of CuTA NSs. H) Quantitative evaluation of the distance between ABC and CEJ. I) Relative fluorescence intensity ofRAW264.7 with different treatments stained by DCFH‐DA. Adapted with permission.^[^ ^16^ ^]^ Copyright 2023, American Chemical Society.

Extensive literature indicates that periodontitis is associated with an excessive accumulation of substances such as ROS and MMPs, leading to continuous destruction and loss of periodontal tissue attachment.^[^ ^190^ ^]^ Consequently, researchers have developed a self‐assembling hydrogel system consisting of copper‐based nanozyme and monostearin glyceride hydrogel, designated as TM/BHT/CuTA (**Figure** **15**A). This system enables the components to adhere to positively charged inflammatory sites via electrostatic attraction and undergo hydrolysis in response to elevated MMP levels during periodontitis, facilitating the on‐demand release of CuTA nanozyme. The released CuTA nanozyme exhibit antimicrobial and anti‐plaque properties. Additionally, as a metal‐phenol nanozyme, it can eliminate various ROS by mimicking the cascade processes of SOD and CAT. Furthermore, CuTA nanozyme can modulate the polarization of macrophages from the pro‐inflammatory M1 phenotype to the anti‐inflammatory M2 phenotype via the Nrf2/NF‐κB signaling pathway, reducing pro‐inflammatory and enhancing anti‐inflammatory factors, thereby promoting the expression of osteogenic genes. This leads to the alleviation of inflammatory responses and accelerates tissue regeneration, achieving multifaceted effects including antimicrobial activity, on‐demand ROS clearance, anti‐inflammatory properties, and bone regeneration (Figure 15B–I).^[^ ^16^ ^]^

Song et al. designed an injectable nano‐hydrogel PPP‐MM‐S loaded with metformin (Met) for diabetic periodontal bone regeneration therapy. The slow degradation of the hydrogel exposes porous MSN and continuously releases stromal cell‐derived factor‐1 (SDF‐1) and Met. On one hand, SDF‐1 continuously recruits bone marrow mesenchymal stem cells (BMSCs) to the periodontal defect site; on the other hand, targeting the mtETC complex I, Met inhibits cell ATP and DNA synthesis, alleviating inflammation induced by excessive mitochondrial ROS production and restoring the impaired osteogenic potential of BMSCs. This cascade treatment mimicking BMSCs' “recruitment‐osteogenesis” efficiently promotes periodontal bone regeneration. However, since MSNs themselves can promote osteogenesis, we believe that the periodontal bone regeneration results confirmed by this study should not be solely attributed to Met's anti‐inflammatory effects, and further exploration of its osteogenic mechanisms is warranted.^[^ ^191^ ^]^

Recently, researchers have developed a mitochondria‐targeted nanomotor that responds to ROS, catalyzing amino acid generation of nitric oxide (NO). While clearing excessive ROS, it also provides itself with the driving force to penetrate deep tissues, allowing the drug to accumulate in deeply damaged areas, significantly promoting stem cell reactivation and proliferation, thus achieving tissue regeneration.^[^ ^192^ ^]^ This method, utilizing the transformation properties of ROS clearance/NO generation to provide differentiation and proliferation stimuli for stem cells to promote tissue repair, deserves further investigation. Additionally, studies have shown that the accumulation of ROS and DNA damage beyond a threshold within cells leads to increased senescent cells and failure of tissue regeneration function. By reducing the number of senescent cells to decrease their inflammatory secretion, stem cell proliferation can be restored and tissue regeneration promoted.^[^ ^193^ ^]^ Therefore, developing drugs targeting ROS chemical reactions to clear senescent cells and restore the differentiation potential of stem cells may serve as a novel approach to promote tissue regeneration. Although many anti‐aging nanoparticle systems have been developed to eliminate senescent cells, there are still few reports on nanodrug research promoting defective tissue regeneration through this method, necessitating further exploration in the future.

## 7 Summary and Prospects

**Figure 16:**
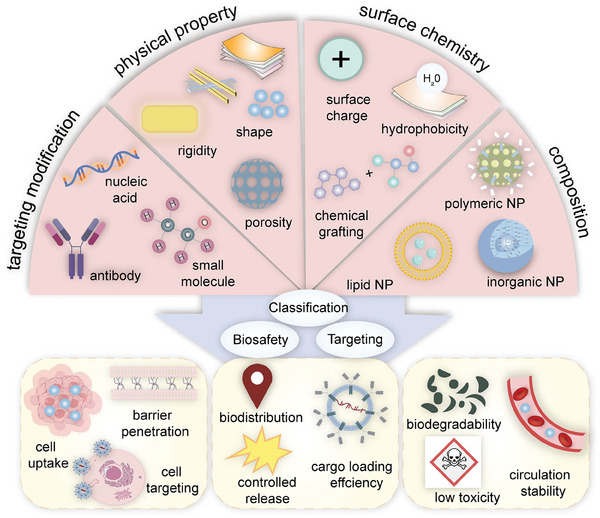
Enhancing nanomaterial diversity, safety, and targeting capabilities through various approaches including surface chemistry, targeted modification, physical properties enhancement, and composite material integration.

In recent years, the emergence of ROS science and significant strides in nanotechnology have paved the way for the development of various ROS‐based nanomaterials designed to modulate the redox state within biological systems. These advancements hold vast application prospects and developmental potential in the biomedical field. This paper offers an exhaustive review of the latest research progress and applications of ROS‐based nanomodulators within the oral cavity domain. Notably, nanomedicines can effectively address a spectrum of oral diseases by either restoring or amplifying oxidative stress levels through diverse mechanisms of action. Despite the notable progress in the application of ROS‐based nanotherapy in dentistry, there remains a pressing need for unified and clear standards for the design and classification of ROS‐based nanomedicines. This gap presents a significant opportunity for future research and development. The establishment of such standards will be instrumental in advancing the field and ensuring the safety and efficacy of these innovative therapeutics. (**Figure** **16**).

First, regarding classification, although the types of nanoplatforms regulating ROS reactions currently cover most of the redox reaction networks, correcting some complex oxidative stress disorders remains challenging. To expand the variety of ROS‐based nanomaterials, it is advisable to consider explicit functional modifications (such as physical adsorption, chemical composition modification, etc.), size adjustment, and morphological transformation of existing nanomedicines. From a broader perspective, future development of nanomedicines should be guided by principles of biomimicry and large‐scale production, necessitating further efforts and exploration.

Second, in terms of targeting, the complexity of biological barriers formed by the cross‐effects of ROS and other factors impedes the effective delivery of nanoparticles, thereby reducing therapeutic efficacy. Therefore, designing nanoparticles with high targeting delivery capabilities holds promise for improving the accuracy and efficiency of ROS‐based drug regulation. Current efforts to enhance the targeting specificity of nanomedicines focus on active targeting strategies (such as biomimetic modifications and extracellular vesicles), resulting in drugs that closely mimic real targeting mechanisms, such as various cell membrane‐coated nanoplatforms and nanorobots. Additionally, responding to changes in the microenvironment is another effective design strategy to enhance targeting. Currently, most microenvironment‐responsive NPs primarily target single factors such as enzymes, pH, or ROS, limiting the application of current nanomedicines in environments with mixed pathological conditions. Therefore, to precisely control treatment outcomes, further refinement is needed in the application scope and response effects of these nanomedicines.

Third, concerning safety, current research on nanomedicines primarily focuses on the bidirectional regulation of ROS. However, the specific effects of some novel nanomedicine components on the complex redox metabolism network remain unclear, and there is a lack of effective research data on material safety and biocompatibility, limiting their clinical translation. In practical clinical exploration, many structurally simple and mechanistically clear nanozymes, nanogels, or microspheres are commonly used as drug carriers, promoting predictability and standardization of drug actions and behaviors in vivo. Therefore, biodegradable nanozymes, natural polymer NPs, and free radical scavenging NPs appear promising alternatives. Additionally, it is necessary to develop new microenvironment detection technologies and establish effective pharmacokinetic computational models to enhance the therapeutic matching between drugs and pathological environments. In conclusion, the development of effective and safe ROS‐regulating nanomedicines requires the combined expertise and skills of scholars from different fields to bring about better innovation and applications.

## Conflict of Interest

The authors declare no conflict of interest.

## Author Contributions

X.L. and Y.Z. conceptualized the article. X.L. wrote the first draft of the manuscript with critical input from all authors. W.Y., Y.Z., and D.Y. reviewed and critically revised the manuscript.
